# *Kaempferia parviflora* Extract Alleviated Rat Arthritis, Exerted Chondroprotective Properties In Vitro, and Reduced Expression of Genes Associated with Inflammatory Arthritis

**DOI:** 10.3390/molecules26061527

**Published:** 2021-03-11

**Authors:** Siriwan Ongchai, Natthakarn Chiranthanut, Siriwan Tangyuenyong, Nawarat Viriyakhasem, Patiwat Kongdang

**Affiliations:** 1 Thailand Excellence Center for Tissue Engineering and Stem Cells, Department of Biochemistry, Faculty of Medicine, Chiang Mai University, Chiang Mai 50200, Thailand; siriwan.ongchai@cmu.ac.th; 2 Center for Research and Development of Natural Products for Health, Chiang Mai University, Chiang Mai 50200, Thailand; 3 Department of Pharmacology, Faculty of Medicine, Chiang Mai University, Chiang Mai 50200, Thailand; natthakarn.c@cmu.ac.th; 4 Equine Clinic, Department of Companion Animal and Wildlife Clinic, Faculty of Veterinary Medicine, Chiang Mai University, Chiang Mai 50100, Thailand; siriwan.tangy@cmu.ac.th; 5 The School of Traditional and Alternative Medicine, Chiang Rai Rajabhat University, Chiang Rai 57100, Thailand; nawaratv@gmail.com; 6 Musculoskeletal Science and Translational Research Center, Department of Orthopedics, Faculty of Medicine, Chiang Mai University, Chiang Mai 50200, Thailand

**Keywords:** anti-arthritis, anti-inflammation, chondroprotection, inflammatory joint disease, *Kaempferia parviflora*

## Abstract

*Kaempferia parviflora* Wall. ex Baker (KP) has been reported to attenuate cartilage destruction in rat model of osteoarthritis. Previously, we demonstrated that KP rhizome extract and its active components effectively suppressed mechanisms associated with RA in SW982 cells. Here, we further evaluated the anti-arthritis potential of KP extract by using multi-level models, including a complete Freund’s adjuvant-induced arthritis and a cartilage explant culture model, and to investigate the effects of KP extract and its major components on related gene expressions and underlying mechanisms within cells. In arthritis rats, the KP extract reduced arthritis indexes, with no significant changes in biological parameters. In the cartilage explant model, the KP extract exerted chondroprotective potential by suppressing sulfated glycosaminoglycans release while preserving high accumulation of proteoglycans. In human chondrocyte cell line, a mixture of the major components equal to their amounts in KP extract showed strong suppression the expression of genes-associated inflammatory joint disease similar to that of the extract. Additionally, KP extract significantly suppressed NF-κB and MAPK signaling pathways. The suppressing expression of necroptosis genes and promoted anti-apoptosis were also found. Collectively, these results provided supportive evidence of the anti-arthritis properties of KP extract, which are associated with its three major components.

## 1. Introduction

Arthritis is defined as joint inflammation that is generally accompanied by cartilage and joint damage [1]. Two of the most common forms of arthritis are osteoarthritis (OA) and rheumatoid arthritis (RA) [1]. They share some similar features, including joint pain, inflammation, and cartilage breakdown [2]. However, these arthritis forms can be differentiated by their clinical appearance and molecular mechanism of pathogenesis. A key factor of OA pathogenesis is chronic low-grade joint inflammation, which contributes to gradual progression of cartilage destruction [3]. RA involves the systemic autoimmune disorders targeting the synovial tissue lining of the joint capsule that lead to chronic high-grade systemic inflammation and progressive damage to cartilage and bone, which results in permanent joint disability [1].

Several pro-inflammatory cytokines have key roles in the pathogenesis of OA and RA and include interleukin (IL)-1β, tumor necrosis factor (TNF)-α, IL-6, and IL-17A [4,5]. Upregulation of the production of these cytokines in these arthritis forms has been well reported [4,5], which causes joint inflammation and contributes to activation of the expression of tissue-degrading enzymes, especially matrix metalloproteinase (MMP)-13. These enzymes need zinc ion as a co-factor. ZIP8, a zinc transporter protein that is reported to increase in arthritis, facilitates accumulation of intracellular labile zinc [6]. In addition, the high levels of pro-inflammatory cytokines in OA cause activation of the apoptotic process of chondrocytes [7] and production of synoviocytes, the key cells in the RA pathogenesis, to increase resistance against cell death [8]. Upregulation of these pro-inflammatory cytokines and the consequent factors involves selective activation of intracellular signaling pathways, including transcription factor nuclear factor kappa B (NF-κB) and mitogen-activated protein kinase (MAPK) [4,5]. Selective suppression of the involved signaling pathway is one of the targets of a therapeutic approach to arthritis.

The pharmacological treatment of OA and RA usually involves anti-inflammatory drugs to reduce symptoms of pain and inflammation, especially steroid and nonsteroidal anti-inflammatory drugs [2,9]. Natural products derived from plants have been considered to be alternative treatments of arthritis from ancient times to the present. *Kaempferia parviflora* Wall. ex Baker (KP), an herb belonging to the Zingiberaceae family, has been reported to exert anti-arthritis activity. In a rat model of OA induced by monoiodoacetic acid, a KP root powder extract reduced cartilage lesions, and its active components, 5,7,4′-trimethoxyflavone (T) and 5,7-dimethoxyflavone (D), suppressed expression of cartilage-degrading enzymes and MMPs [10]. Another active compound of KP, 5,7,3′,4′-tetramethoxyflavone (Te), has been reported to ameliorate pro-inflammatory cytokines levels in knee synovial fluid of OA induced rats by transection of anterior cruciate ligaments [11]. This active component was found to reduce the apoptotic rate of chondrocytes [11,12]. We recently reported that a KP extract and its active components, D, T, and 3,5,7,3′,4′-pentamethoxyflavone (P), downregulated mechanisms associated with RA in a SW982 cell culture model by selective suppression of the p38/STAT1 and STAT3 pathways [13].

The present study aimed to provide additional evidence of the anti-arthritis potential of KP by using multi-level models, including a complete Freund’s adjuvant (CFA)-induced rat arthritis model and a cartilage explant culture model to show chondroprotective potential, and to investigate molecular mechanisms and signaling pathways within cells that are related to arthritis, especially expressions of pro-inflammatory cytokines genes and cell death-associated genes.

## 2. Results

### 2.1. Attenuating Effect of KP Extract on Rat Arthritis Induced by CFA

**Figure 1:**
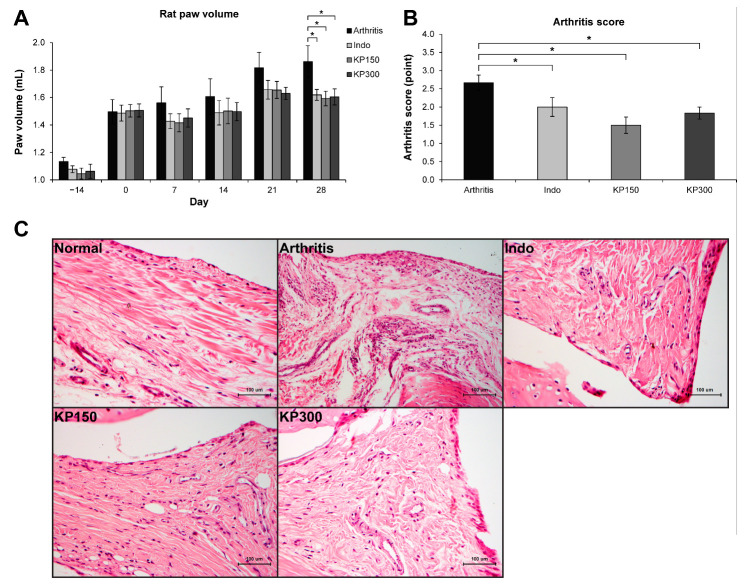
KP extract attenuates CFA-induced rat arthritis. (A) A graph shows an increase in the right paw volume of all groups on day 0, indicating the onset of an experimental RA induced by CFA injection. Then, the induced rats were fed with 3 mg/kg/day of indomethacin (Indo), 150 mg/kg/day of KP extract (KP150), or 300 mg/kg/day of KP extract (KP300) for another 28 days. The results show that Indo, KP150, and KP300 relatively decreased rat paw edema since day 7 and significantly on day 28, compared to the arthritis control group (Arthritis). (B) A significant reduction of arthritis severity scores, which were evaluated on day 28, was observed in the arthritis rats treated with Indo, KP150, and KP300, when compared with the arthritis control. (C) The H&E-Scheme 150. and KP300 groups. Results represent mean ± SEM of 3 rats (normal group) or 6 rats (treated groups). One-way ANOVA followed by the post hoc Fisher’s test was used to analyze the statistical difference (* *p* < 0.05).

The CFA-induced rat arthritis model was adopted to evaluate the anti-inflammatory effects of the KP extract in animals. The positive control group was animals who were orally administered indomethacin. Figure 1A shows an increase in rat paw volume in all groups on the first day of the treatments (day 0), indicating the onset of RA. The decreasing trend of rat paw edema in the three arthritis rat groups fed with indomethacin (3 mg/kg/day) and KP extract (150 and 300 mg/kg/day) was observed throughout the study period and showed significant differences in paw volume relative to those in the arthritis controls in the last week. The inhibitory activities of the indomethacin-treated group were not significantly different from those of the arthritis rats fed with KP extract. Compared with the arthritis rats control, the arthritis rats treated with indomethacin and KP extract (150 and 300 mg/kg/day) showed significant reductions in arthritis severity scores by 25.0%, 43.8%, and 31.3%, respectively (Figure 1B).

The histopathological analysis of synovial membranes in the arthritis group clearly illustrated the characteristics of arthritis relative to those in the normal group (Figure 1C). Thickening of cells was observed along the synovial lining. There were an increased number and enlarged size of blood vessels concomitantly with highly infiltrated inflammatory cells, which were ubiquitously found in synovial membranes of the arthritis group. The amount and size of blood vessels and inflammatory cells were decreased in the arthritis rats treated with indomethacin and KP extracts.

The synovial membranes of the hind paw joint on H&E-stained sections were used to evaluate levels of synovial inflammation by using Gynther’s system (Table 1). The untreated group had the lowest average scores of synovial lining layers, vascularity, and presence of inflammatory cells. In the arthritis group, the levels of synovial hyperplasia, proliferation of blood vessels, and infiltration of inflammatory cells, were observed. These levels were reduced in the groups treated with indomethacin and KP extracts, except for the synovial lining layers’ score, which was still high in the indomethacin group.

Effects of the treatments on biological parameters, such as organ weights, hematological features, and blood chemistries, in the treatment of arthritis rats were evaluated in parallel with those of indomethacin (Table 2). In contrast with the normal control, the arthritis rats treated with indomethacin showed a significant increase in spleen weight of 42.9% and some changes in hematological parameters, including reduction in red blood cell, hemoglobin, and hematocrit by 24.8%, 22.0%, and 18.3%, respectively, but no changes in other blood chemistry markers were observed. The platelet count in this group was increased by 53.8% over that of the normal control, even though this number is still within the normal range. In arthritis rats treated with KP extract, the significant changes in biological parameters as mentioned above were not observed, except for a reduction in kidney weight by 14.6% in arthritis rats treated with KP 150 mg/kg/day.

### 2.2. Chondroprotecting Effects of KP Extract on Cartilage Explant Degradation

**Figure 2:**
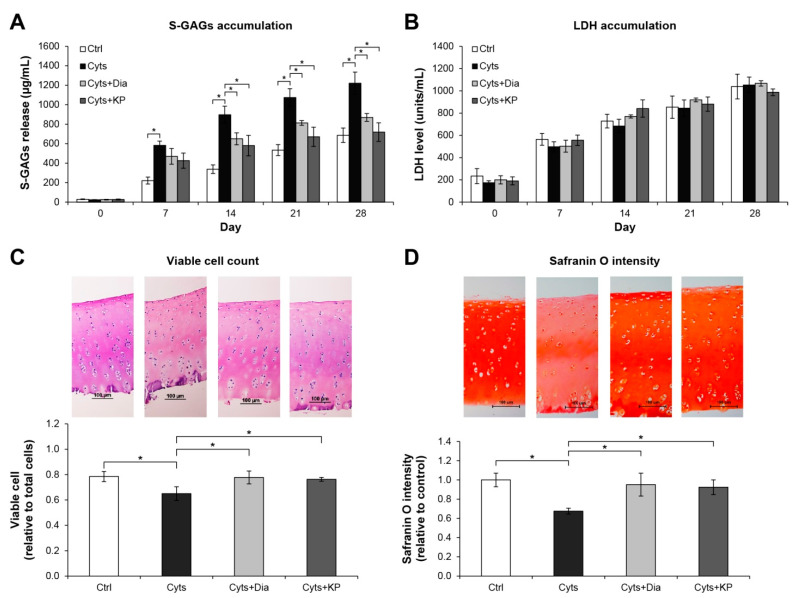
KP extract protects cartilage explant degradation. Porcine cartilage explant degradation was stimulated by a combination of 2 ng/mL of IL-1β and 4 ng/mL of IL-17A (Cyts) with or without KP extract (25 μg/mL) or 50 μM of diacerein (Dia) for 28 days. The control (Ctrl) explants were left untreated. A significant increase in accumulation of released S-GAGs induced by the combined cytokines was significantly decreased when co-treated with KP extract or diacerein (**A**). The non-significant differences in lactate dehydrogenase (LDH) accumulation in the culture media among all treatment groups and the control indicate the non-cytotoxic effects of the reagents and KP extract on the cartilage cells (**B**). Histopathological examination of cartilage disc (day 28) illustrates restoring of the viable cell numbers (**C**) and safranin O intensity (**D**) in co-treatment of the combined cytokines with KP extract or diacerein. Results represent mean ± SD of three independent experiments. One-way ANOVA followed by the post hoc Tukey multiple comparison test was used to analyze the statistical difference (* *p* < 0.05).

The long-term culture model of porcine cartilage degradation induced by the combination of the pro-inflammatory cytokines, IL-1β, and IL-17A, was used to investigate the chondroprotective potential of the KP extract. A significant increase in accumulation of released S-GAGs was observed in the combined cytokines-treated group relative to that in the untreated control (Figure 2A). This increase in released S-GAGs was significantly decreased when co-treated with KP extract, which was similar to the result in the diacerein-treated group, the anti-arthritis drug that was used as a positive control. Histological examination of the cartilage disc of the combined cytokines-treated group revealed a reduction in cell numbers and safranin O intensity by 17.2% and 26.0%, respectively, relative to the levels in the untreated control (Figure 2C,D). These levels were significantly improved when co-treated with KP extract or diacerein. Figure 2B shows the non-significant differences in lactate dehydrogenase accumulation in the culture media among all treatment groups and the control.

### 2.3. Suppressing Effect of KP Extract and Its Major Components on Arthritis-Related Gene Expressions and Signaling Pathways in SW1353 Cells

**Figure 3:**
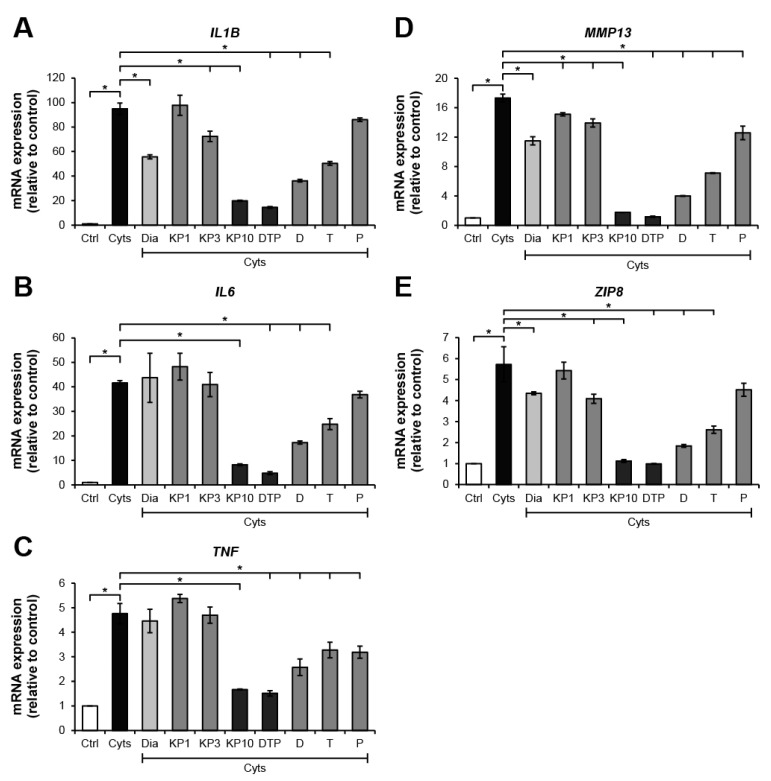
KP extract and its major components suppress arthritis-related gene expressions in SW1353 cells. SW1353 was activated by a combination of 2 ng/mL of IL-1β and 4 ng/mL of IL-17A (Cyts) for 24 h with or without diacerein (Dia; 50 μM), KP extract (1, 3, and 10 μg/mL), 5,7-dimethoxyflavone (D; 3.3 μg/mL), 5,7,4′-trimethoxyflavone (T; 2.6 μg/mL), 3,5,7,3′,4′-pentamethoxyflavone (P; 2.2 μg/mL) or a mixture of the three major compounds (DTP), which is equal to the estimated proportions in the KP extract at 10 μg/mL (D: T: P = 3.3: 2.6: 2.2 μg/mL). Ctrl is an untreated control. The dramatic increase in mRNA expressions of the arthropathy-associated inflammatory cytokines genes, *IL1B* (**A**), *IL6* (**B**), and *TNF* (**C**), cartilage-degrading enzyme, *MMP13* gene (**D**), and zinc transporter 8 (*ZIP8*) gene (**E**), was found when the cells were treated with the combined cytokines. These increased expressions were significantly suppressed by KP extract at a concentration of 10 μg/mL, which was similar to the effect of the mixture of the three major compounds that equal to their amounts in 10 μg/mL KP extract. Results represent mean ± SD of three independent experiments. One-way ANOVA followed by the post hoc Tukey multiple comparison test was used to analyze the statistical difference (* *p* < 0.05).

The chondrosarcoma cell line, SW1353, stimulated by the combined cytokines, IL-1β and IL-17A, was selected to evaluate the effects of KP extract and its major components at a cellular level. Figure 3 illustrates the high increase in mRNA expressions of the arthropathy-associated inflammatory cytokines genes, *IL1B*, *IL6*, and *TNF*, when the cells were treated with the combined cytokines. The cartilage-degrading enzyme, *MMP13*, and the zinc transporter gene, *ZIP8*, were also strongly activated. These were significantly suppressed by KP extract at a concentration of 10 ng/mL. The standard compounds, D, T, and P, which were found to be the major components of the KP extract (Figure S1), were prepared at concentrations of 3.3, 2.6, and 2.2 μg/mL, respectively. These were equal to the estimated proportions in the KP extract at 10 μg/mL. Among these components, D showed the strongest inhibitory effects on the expression of these genes. Treatment of the cells with the combination of these components strongly counteracted induction of the combined cytokines similar to the effect of the KP extract at 10 μg/mL. Diacerein showed weaker suppressive effects on the expressions of *IL6* and *TNF* than the standard components and the KP extract at 10 μg/mL.

**Figure 4:**
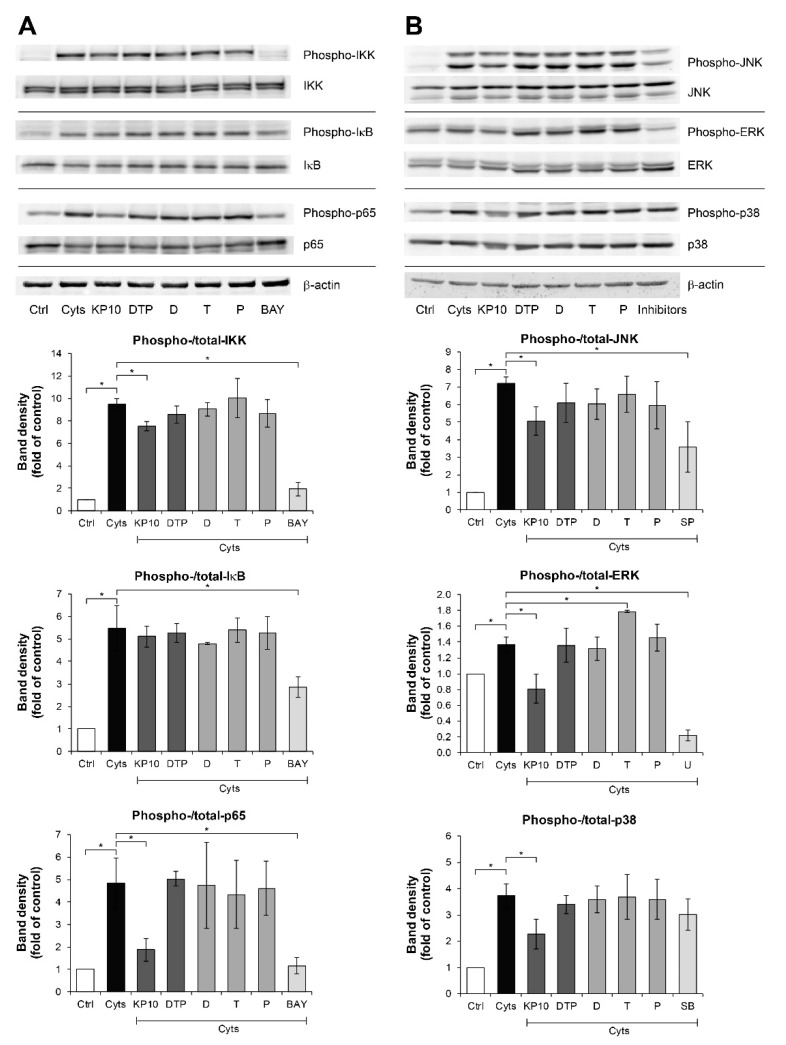
KP extract suppresses the NF-κB and MAPK pathways in SW1353 cells. SW1353 cells were pretreated for 2 h with KP extract (10 μg/mL), 5,7-dimethoxyflavone (D; 3.3 μg/mL), 5,7,4′-trimethoxyflavone (T; 2.6 μg/mL), 3,5,7,3′,4′-pentamethoxyflavone (P; 2.2 μg/mL) and a mixture of the three major compounds (DTP), which equal to the estimated proportions in the KP extract at 10 μg/mL (D: T: P = 3.3: 2.6: 2.2 μg/mL), and signaling inhibitors, including BAY11-7082 (BAY; 20 µM), SP600125 (SP; 10 µM), U0126 (U; 5 µM), and SB203580 (SB; 1 µM). The cells were then co-treated with IL-1β 2 ng/mL and IL-17A 4 ng/mL (Cyts) for 25–30 min. The untreated group was left as a control (Ctrl). The representative immunoblots against NF-κB and MAPK signaling molecules are shown in (**A**) and (**B**), respectively. Bar graphs are mean ± SD of two independent experiments in the ratios between the band intensities of phosphorylated form and total form of each condition. One-way ANOVA followed by the post hoc Fisher’s test was used to analyze the statistical difference (* *p* < 0.05). The band density for each sample was normalized to β-actin. The full-length blot images are provided in Supplementary Data (Figures S6 and S7).

We further investigated the molecular actions of the KP extract and its major components in signal transductions of the NF-κB and MAPK pathways, which are involved in the pathogenesis of arthritis. These pathways were activated by treating SW1353 cells with IL-1β and IL-17A and were suppressed by their specific inhibitors. Regarding the NF-κB pathway (Figure 4A), in comparison with the effect observed in the cytokines-treated groups, phosphorylation of IKK and p65 was significantly suppressed by the KP extract by 20.3% and 61.2%, respectively. Consistently, phosphorylation of JNK, ERK, and p38 MAPK signaling molecules were significantly reduced by 29.8%, 40.6%, and 39.0%, respectively, by the KP extract (Figure 4B). The mixture of its major components slightly decreased JNK phosphorylation by 15.7%; however, it did not affect the phosphorylation of NF-κB and MAPK pathways.

### 2.4. Protecting Effects of KP Extract against Cell Death Pathways

**Figure 5:**
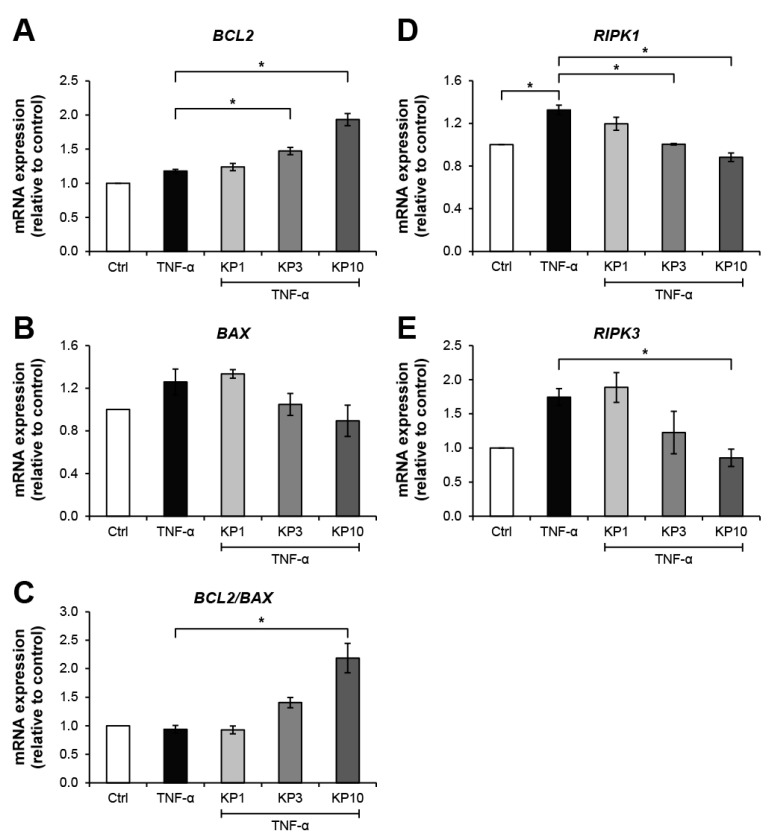
KP extract suppresses the expressions of necroptosis-related genes but promotes the anti-apoptosis gene. SW1353 cells were pretreated with TNF-α 10 ng/mL for 2 h to trigger cell death-associated gene expression, followed by a co-treatment with KP extract (1, 3, and 10 μg/mL) for 24 h. The untreated group was left as a control (Ctrl). In KP extract co-treated groups, an increasing trend of anti-apoptosis process was observed from the upregulation of *BCL2* gene expression (**A**) and the suppression of *BAX* gene expression (**B**), which contributed to the increasing ratio of *BCL2*:*BAX* gene expressions (**C**). The upregulation of necroptosis-related gene expression, *RIPK1* (**D**) and *RIPK3* (**E**), induced by TNF-α was significantly suppressed by the KP extract at 10 μg/mL. Results represent mean ± SD of three independent experiments. One-way ANOVA followed by the post hoc Tukey multiple comparison test was used to analyze the statistical difference (* *p* < 0.05).

**Figure 6:**
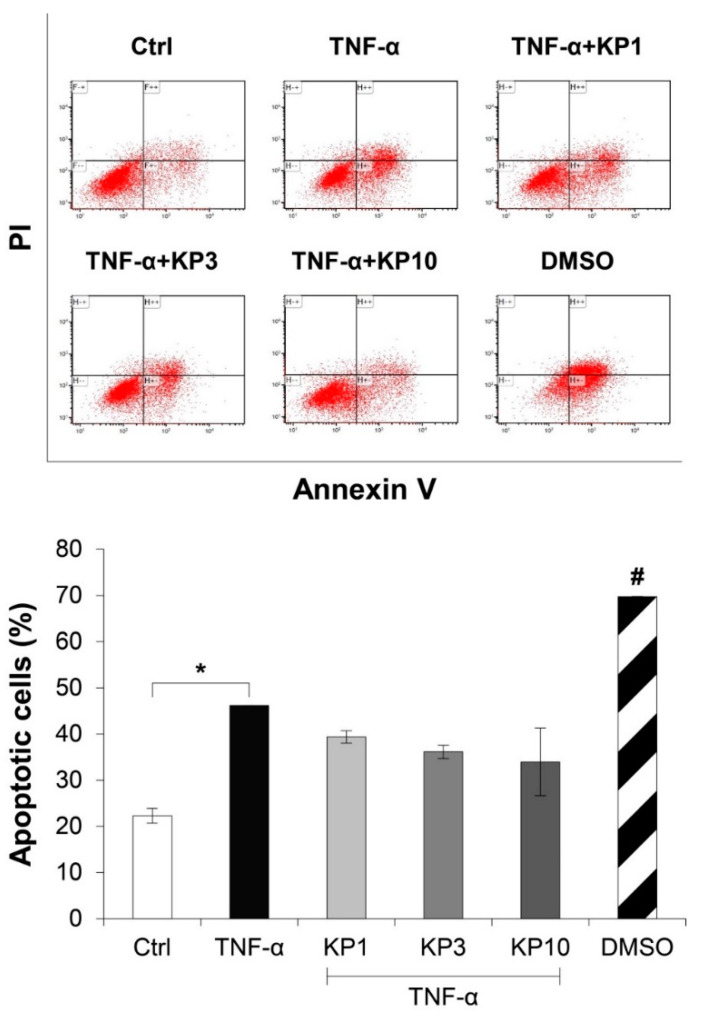
KP extract reduces the number of apoptotic cells. SW1353 cells were pretreated with TNF-α 50 ng/mL for 2 h and followed by co-treatment with the KP extract (1, 3, and 10 μg/mL) for another 48 h. DMSO (2.5%) was used as a positive control. The untreated group was left as a control (Ctrl). The number of apoptotic cells was counted by flow cytometry. The significant increase in apoptotic cells induced by TNF-α is counteracted by the KP extract, lead to a decreasing trend of the apoptotic cell number. Results represent mean ± SD of two independent experiments. One-way ANOVA followed by the post hoc Tukey multiple comparison test was used to analyze the statistical difference (* *p* < 0.05 and ^#^ *p* < 0.001 compared to the untreated control).

Figure 5 illustrates the effects of the KP extract on the expressions of genes related to cell death. The TNF-α-treated group showed slightly elevated expressions of *BCL2* (Figure 5A), an anti-apoptosis gene, together with *BAX* (Figure 5B), a pro-apoptosis gene, which led to no change in the ratio of *BCL2*:*BAX* gene expressions (Figure 5C). In co-treatment with the KP extract, a trend of increasing ratio of *BCL2*:*BAX* gene expressions was observed and reached significant levels compared with the untreated control by treatment with the KP extract at 10 μg/mL. Regarding the expressions of genes associated with the necroptosis pathway, TNF-α caused significant elevation of the gene expressions of receptor interacting protein kinase 1 (*RIPK1*) and *RIPK3*, which were significantly suppressed by the KP extract at 10 μg/mL (Figure 5D,E). The significant increase in apoptotic cells as evaluated by flow cytometry was found when the cells were treated with TNF-α at 50 ng/mL or DMSO, the positive control groups (Figure 6). Co-treatment of this cytokine with the KP extract led to a decreasing trend in apoptotic cells, even though there was no significant difference when compared with the effect in the TNF-α-treated group.

## 3. Discussion

Recently, a number of studies have demonstrated the potential activities of KP extract and its major constituents, which are beneficial in the treatments for various diseases [15,16]. Nevertheless, there have only been a few reports in the last decades on the anti-arthritis potential of KP extract [13,15]. The present study provides more evidence to support the anti-arthritis potential of the rhizome extract of KP in a CFA-induced arthritis rat model in addition to demonstrating its in vitro chondroprotective activities that were assessed in a cartilage explant model. At the cellular level, the KP extract showed suppressive effects on expressions of genes involved in inflammatory arthritis and cartilage degradation. The combination of the three major methoxyflavones (D, T, and P) of the ethanolic extract that was prepared at concentrations equivalent to their concentrations in the KP extract clearly demonstrated synergistic effects by downregulating the expressions of genes associated with arthritis in a manner similar to the effects of the KP extract. Regarding the signaling pathways involved in the anti-arthritis effects, the KP extract showed strong selective suppression of the phosphorylation of some proteins, which involves the NF-κB and MAPK pathways. In addition, the extract appeared to have protective activities against TNF-α-induced apoptosis and necroptosis.

The ethanolic extract of the KP rhizome, which was evaluated by HPLC analysis, showed three major methoxyflavones: D, T, and P at proportions of 36.74%, 26.72%, and 30.91%, respectively (Figure S1). These proportions were similar to the findings of a previous report [17]. It has been reported that these three components were absorbed into the bloodstream within 1 to 2 h after oral administration of 750 mg/kg of a KP extract followed by detecting these compounds in various organs, including the liver, kidney, lung, testes, and brain, and were finally excreted with half-lives of 3 to 6 h [17]. A chronic toxicity study was conducted in Wistar rats by oral administration of KP extract for 6 months with doses ≤ 500 mg/kg/day, which is equivalent to 100 times the dose for human use [18]. The results showed significantly lower body weight with slight changes in hematological parameters. However, our results revealed non-significant change in the biological parameters. The average weight of rats in all groups showed an increasing trend during the experimental period but the difference was not significant (Figure S2). These results indicate a possible safe dose range of the KP extract for human use. As reported in a systemic review, there were no adverse health effects in humans by using this plant up to 1.35 g/day [15].

In the present study, inflammation in multiple joints of rats injected with CFA was successfully achieved in the arthritis rat model, which was similar to findings previously reported [19]. Our results showed induction of joint swelling with high accumulation of lymphocyte infiltration, but signs of cartilage destruction were not clearly observed in this study. This arthritis rat model is widely used because the RA rat shares common symptoms with those of humans with RA, including polyarticular inflammation, synovial hyperplasia, and cartilage degradation [20]. Throughout the experimental period, arthritis rats treated with indomethacin (3 mg/kg/day) and KP extract (150 and 300 mg/kg/day) demonstrated a decreasing trend in paw volume that reached significantly different levels in the last week relative to the paw volumes in the arthritis control group. This finding was supported by the significant reduction in arthritis scores in the arthritis rats treated with the KP extract and indomethacin.

These results suggested that the KP extract at concentrations of 50- and 100-fold indomethacin exerted similar anti-arthritis potential. Indomethacin is a nonsteroidal anti-inflammatory drug that is used to relieve fever, pain, and swelling. This drug was reported to reduce prostaglandin E2 (PGE2) and TNF-α levels in carrageenan-induced acute inflammation in rats [21]. A recent report on the use of 2.5 mg/kg/day indomethacin in CFA-induced arthritis in rats did not show significant changes in the hematological profile relative to those in the arthritis control [22]. However, our present study revealed that the oral dosage of 3 mg/kg/day indomethacin significantly enhanced hypersplenism and some hematological parameters.

The histopathological changes in the joint tissue sections were examined by H&E staining. The synovitis features that were increased in the joints of the arthritis rats, including synovial hyperplasia, proliferation of blood vessels, and infiltration of inflammatory cells, were improved in both KP groups and the indomethacin group. Nevertheless, the KP extract did not show anti-arthritis in a concentration-dependent manner. Although treatments with the higher dose of KP extract at 300 mg/kg/day did not provide better results than those at the dose at 150 mg/kg/day, the results indicate the potent anti-inflammatory activities of the KP extract in an animal model. These results are supported by those of our previous study, which used a synovial sarcoma cell line, SW982 [13]. The previous study results showed that the KP extract and its three major components, D, T, and P, suppressed several factors associated with RA, including expression of pro-inflammatory cytokines, inflammatory mediators, and cartilage-degrading enzymes. The extract downregulated genes of autophagosome and necroptosome formations and suppressed cell migration, but it neither dissolved apoptosis resistance nor altered the cell cycle. Although the current study provided strong evidence of a potent anti-inflammatory effect of the KP extract, the chondroprotective potential of the KP extract under joint inflammation conditions was not observed in this animal model.

The cartilage explant model was used to evaluate the chondroprotective potential of KP in vitro. This model simulates the three-dimensional environment of native cartilage, which is useful for basic screening of the chondroprotective potential of anti-arthritis drug candidates [23]. The loss of cartilage matrix molecules was shown by the significantly decreasing S-GAGs levels in the culture media, which was consistent with the high remaining content of proteoglycans, as shown by safranin O-stained tissue sections. The reduction in viable cell numbers caused by cytokines treatment was reversed when the cells were co-treated with the extract. These results suggest the chondroprotective potential of a KP extract against cytokines-induced cartilage degradation. This protective effect might be related to the suppressive effects of the KP extract on cellular mechanisms associated with cartilage degradation and death of chondrocytes in the explants, which are induced by IL-1β and IL-17A [24,25]. Our results supported those of previous studies on the chondroprotective potential in animal models of the KP extract [10] and Te [11]. LDH levels in the culture media of all treatments were not significantly different than those in the control, which suggested that the changes in all parameters in this study were not caused by the cytotoxic effects of the reagents and KP extract on the cartilage cells.

SW1353, a chondrosarcoma cell line, was used to better understand the protective mechanisms of KP at cellular levels. This cell line has been well established as a representative for chondrocytes in many previous studies [26,27,28]. The SW1353 cell line was reported to be activated by IL-1β and contributed to an increase in the expression of catabolic factors involved in the development of OA and RA, including pro-inflammatory cytokines, TNF-α, IL-6, and IL-8, and a cartilage-degrading enzyme, MMP-13 [29]. These pro-inflammatory mediators, especially TNF-α and IL-6, are well observed to involve RA pathogenesis [30]. Consistent with previous reports, these genes were also significantly upregulated by the combined IL-1β and IL-17A. These genes were suppressed by the KP extract at 10 μg/mL and its major constituents, D, T, and P, at respective concentrations similar to those in the 10 μg/mL KP extract. These results suggest that these three major components are required in the anti-inflammatory mechanism as active secondary metabolites of KP and that they helped mitigate inflammatory indicators in arthritis rats fed with KP extract as evaluated by paw volume, arthritis scores, and synovitis scores.

Among the three major components of KP, D showed stronger inhibitory effects on expressions of the catabolic factors than those of T and P. However, the combined DTP showed much higher inhibitory effects than D, T, and P. A powerful synergistic suppression effect was revealed when the three major components were mixed at concentrations equal to their estimated proportions in a 10 μg/mL KP extract. This similar manner was shown in our previous report using a human synovial sarcoma cell line, SW982, which was induced by a combination of IL-1β and IL-17A [13]. We found that the ethanolic extracts of KP, D, and T significantly downregulated expression of *IL1B*, *TNF*, and *COX2* genes in SW982 cells, whereas P selectively suppressed only the expression of *TNF* gene. The KP extract was not able to reduce expression of *IL6* gene in SW982 cells, whereas the combination of D, T, and P strongly downregulated the high expression of these pro-inflammatory genes in SW982 cells in a manner similar to the suppressive action in SW1353 cells described above. The similar suppressive activities of the mixture and the KP extract suggests that those compounds are the main components of KP and have key roles together in enhancing the biological activities of the extract. Furthermore, the different extraction methods of KP produced various patterns of active constituents, which may have contributed to their particular biological activities. These results suggest that the combination of these active major components could provide a greater benefit over the use of the KP extract for treatment strategies in OA and RA.

MMP-13 is an inducible enzyme stimulated by pro-inflammatory cytokines associated with the pathogenesis of OA and RA. This enzyme is mainly produced by chondrocytes and has a role in the degradation of cartilage matrix molecules, including collagen, proteoglycans, and aggrecan [31]. Productions of other MMPs, which are capable of degrading non-collagen matrix protein, were reported to increase in arthritis [32,33]. These enzymes need zinc ion as their co-factor; therefore, the expressions of zinc transporter proteins, especially ZIP8, which facilitates cellular accumulation of zinc ion, were also elevated in OA [6]. Accordingly, suppressions of production of MMP-13 and ZIP8 have been proposed as targets for treating OA [34,35]. Our present results demonstrated that the KP extract and its three major components, especially in a combination treatment, effectively downregulated expressions of the cartilage-degrading enzyme, *MMP13*, and of *ZIP8* in SW1353 cells, similar to the results of our previous study, which was conducted in an SW982 cell culture model [13]. It has been reported that suppression of *ZIP8* reduced cartilage degradation in animal OA induced by destabilization of medial meniscus surgery [36]. In a rat model of OA, oral administration of a KP extract was found to ameliorate cartilage lesions and suppress IL-1β-induced expression of the cartilage-degrading enzymes, *MMP1*, *MMP3*, and *ADAMTS4*, in human chondrocytes [10]. D and T were suggested to be involved in these mechanisms because they are the main absorbable active components of the KP extract. Accordingly, we postulated that one of the mechanisms related to the chondroprotective activities of KP, which was demonstrated in the cartilage explant culture model, involves effective suppression of MMPs and ZIP8 productions.

At present, the NF-κB and MAPK pathways are known to be involved in the pathogenesis of OA and RA [37,38]. Activation of these pathways has been reported to amplify the expression of pro-inflammatory cytokines and cartilage-degrading enzymes [37,39]. Our previous study in an SW982 synovial cell culture model induced by the same combined cytokines showed that the KP extract selectively suppressed p38 MAPK phosphorylation but not the NF-κB pathway [13]. In the meantime, the present study in SW1353 chondrocyte cell line surprisingly demonstrated that the KP extract exerted potential inhibitory effects on suppression of both NF-κB and MAPK pathways. This suggests that the diverse responses of KP extract to the intracellular inflammatory pathways may depend on specific cell types. Further in-depth investigations in various pathways are suggested to extend in primary chondrocytes and synoviocytes. Besides the KP extract, the combination of the three major compounds solely revealed weak inhibitory effect on these signaling pathways, even though it dramatically downregulated the expression of genes-associated inflammatory joint diseases. This finding suggests that the combined major compounds may take a part in the other crosstalk pathways involved with inflammatory joint diseases, such as JAK/STAT and PI-3K/AKT/mTOR pathways, resulting in the inflammatory processes being finally ameliorated [37]. Therefore, the selective effects of the combined major compounds of KP extract on intracellular signaling pathways involved with joint inflammation should be further elucidated.

Although the elevation of pro-inflammatory cytokines in joint microenvironments was similar in OA and RA, the key cells in these diseases responded in different ways to conditions, especially the process of cell death. Pro-inflammatory cytokines activate apoptotic pathways in chondrocytes, which leads to reduction of chondrocyte numbers in OA cartilage and to cartilage degradation [7]. In RA, chronic inflammation causes persistent elevation of pro-inflammatory cytokines production, which contributes to changes in synoviocytes to a tumor-like phenotype, increased resistance to apoptosis, and expansion of the synovial lining and stromal cells [40,41]. Protection of the inflamed chondrocytes against apoptotic induction while suppressing tumor-like transformation and reverse apoptotic resistance of the inflamed synoviocytes are research targets for development of anti-arthritis drugs [42]. The present study demonstrated induction of the apoptosis and necroptosis processes in chondrosarcoma SW1353 cells by TNF-α, which were altered by the KP extract. Although KP extract effectively altered these processes, it did not influence the cell cycle (Figure S3). These results show the protective potential of KP against cell death processes in this chondrocyte cell line. As discussed above, these results support the chondroprotective activities of KP in a cartilage explant model as shown by restoration of viable chondrocyte numbers. The chondroprotective activities of Te by alleviating ER stress-promoted chondrocytes apoptosis by suppressing the expression of GSK-3β have been previously reported [12].

Taken together, this study clearly demonstrated the anti-arthritis potential of the KP extract in a rat model induced by CFA activation. The in vitro models of cartilage explant and the chondrosarcoma cell line culture provided results that support the involvement of anti-arthritis mechanisms of the KP extract and its major components, D, T, and P, including chondroprotection, anti-inflammation, and chondrocytes protection. Therefore, we consider that these KP extract effects were caused by the synergistic actions of the three major components. Accordingly, we propose that the combination of these major components could possibly be used in therapeutic strategies for treatment of OA and RA.

## 4. Materials and Methods

### 4.1. Chemical Reagents

CFA, Dulbecco’s Modified Eagle’s Medium (DMEM), fetal bovine serum (FBS), and other cultured reagents were obtained from Gibco (Grand Island, NY, USA). Recombinant human IL-1β and IL-17A were purchased from ProSpec Protein Specialist (Ness-Ziona, Israel). Standard compounds, D (purity, 99%), T (purity, 99%), Te (purity, 99%), and 3,5,7,3′,4′-pentamethoxyflavone (P; purity, 95%) were purchased from INDOFINE Chemical Company (Figure S8). Indomethacin was purchased from Sigma-Aldrich. Pentobarbital (Nembutal) was obtained from Ceva Santé Animale, France. BAY11-7082, SB203580, and SP600125 were obtained from Calbiochem, and U0126 was purchased from Cell Signaling Technology.

### 4.2. Plant Extraction and Preparation

An ethanolic extraction of KP (voucher specimen number 023202) was performed as previously described [13], and gave a yield of 4.91% (*w*/*w*). Briefly, dried rhizome powder was macerated in 95% ethanol for three days and then re-extracted twice. Pooled supernatants were evaporated to obtain the crude extract, which was kept at −20 °C. The high-performance liquid chromatography (HPLC) profile of the KP extract is shown in Figure 1. The major components were D (36.74%), T (26.72%), and P (30.91%). The extract was dissolved in 5% polysorbate 80 (Tween 80; Merck Millipore) for the animal experiments and in 100% dimethyl sulfoxide (DMSO; VWR), in which the final concentrations were less than 0.05%, for treatments of cartilage explant and cell cultures.

### 4.3. Complete Freund’s Adjuvant-Induced Rat Model

Male Sprague-Dawley rats weighing 140–160 g were purchased from Nomura Siam International Co., Ltd., Bangkok, Thailand. The rats were housed in a cage under environmentally controlled conditions of 24 °C ± 1 °C and a 12-h light/dark cycle with chow pellets and water ad libitum. The experimental procedures were performed as previously described [43] and approved by the Animal Ethics Committee of the Faculty of Medicine, Chiang Mai University, Thailand (protocol approval number: 13/2561). All animal experiments were performed in accordance with relevant guidelines and regulations. After acclimatization for 1 week, the rats were injected with 0.1 mL of CFA into the subplantar surface of the right paw. Non-injected rats were used as a normal control (3 rats). After 14 days, the arthritis rats were selected and randomly divided into four groups (6 rats per group) as follows:

Group 1 = arthritis control group, received 5% Tween 80 at a dose of 5 mL/kg;

Group 2 = reference group, received indomethacin at a dose of 3 mg/kg;

Groups 3 and 4 = test groups, received KP extract at doses of 150 and 300 mg/kg, respectively.

The rats were orally administered test substances once daily at the same time for 28 days, and their body weight and right paw volume were measured by using a plethysmometer (model 7140; Ugo Basile, Varese, Italy) every 7 days. On day 28, the rats were euthanatized by an intraperitoneal injection of pentobarbital (50–90 mg/kg). Blood samples were collected by cardiac puncture to assess hematological parameters and blood chemistry, including glucose, liver function test, and kidney function test, at the Small Animal Hospital, Faculty of Veterinary Medicine, CMU, Thailand. Liver, spleen, kidney, and thymus were weighed using an electronic digital scale (Ohaus, Parsippany, NJ, USA).

### 4.4. Assessments of Arthritis Severity Scores

Arthritis severity evaluation of the right paw was assessed in a blinded scenario on the last week by a veterinarian. The severity of arthritis was assessed using a scale of 0–4 according to the Nature Protocol scoring system [44] as follows: 0 = normal; 1 = mild swelling of tarsals or ankle joint; 2 = mild swelling from the ankle to the tarsals; 3 = moderate swelling from the ankle to metatarsal joints; 4 = severe swelling, including the ankle, foot, and digits.

### 4.5. Histopathological Examination

Right paws were cut after sacrifice and preserved in 4% (*v*/*v*) formaldehyde (RCI Labscan) to perform paraffin embedding. Blinded histopathological assessments of synovitis using Gynther′s system [14] was performed under the guidance of a pathologist. Hematoxylin-eosin (H&E)-stained sections (5 μm thickness) were scored using the following parameters. Synovial lining layers were assessed on the degree of synovial hyperplasia using the following grades: 0 = normal (1–2 cell layers); 1 = slight hyperplasia (2–3 cell layers); 2 = moderate hyperplasia (3–5 cell layers); 3 = strong hyperplasia (more than 5 cell layers). The scorings of number and size of blood vessels in synovium were scored as 0 = less than 5 of small blood vessels/mm^2^; 1 = 5–10 of small blood vessels/mm^2^; 2 = 11–15 of small or medium blood vessels/mm^2^; 3 = more than 15 of small, medium, and large blood vessels/mm^2^. Presence of inflammatory cells in synovium was scored as 0 = less than 2 of inflammatory cells/mm^2^; 2 = 3–10 of inflammatory cells/mm^2^; 5 = 11–50 of inflammatory cells/mm^2^; 10 = more than 50 of inflammatory cells/mm^2^. An inverted confocal microscope (Zeiss Axio, Jena, Germany, with IMTcamCCD5 PLUS, IMT i-Solution, Inc., Vancouver, BC, Canada) was used to capture and scale the histopathological images at 40x magnification.

### 4.6. Porcine Cartilage Explant Model

Fresh feet of pigs (*Sus scrofa domesticus*) 20–24 weeks old were obtained from the local market, Chiang Mai, Thailand. Cartilage discs were dissected and cultured in a 24-well plate at three pieces (approximately 30–35 mg total) per well following a previous work [29]. Cartilage explants were pretreated with 2 ng/mL IL-1β combined with 4 ng/mL IL-17A for 12 h followed by co-treatments with KP extract and diacerein (Artrodar; an anti-arthritis drug) for 28 days, which replaced the treatments every 7 days. Cartilage viability test was assessed by performing a lactate dehydrogenase (LDH) assay, as previously described [45]. A release of LDH into culture media was measured weekly and accumulated levels were analyzed until the end of the experiment.

### 4.7. Assessments of Cartilage Degradation

Sulfated glycosaminoglycans (S-GAGs) released into the culture media indicated destruction of cartilaginous extracellular matrix, which was weekly measured using dimethylmethylene blue assay, as described previously [46]. On the last date of the treatments, cartilage discs were fixed in 4% (*v*/*v*) formaldehyde and underwent paraffin-embedded sectioning. The cartilage sections were then stained with H&E and safranin O dyes. The viability of chondrocytes indicated by the number of lacuna cells per total cells was observed on H&E-stained sections. The remaining proteoglycans in cartilage discs were evaluated by using ImageJ software to measure the color intensity of safranin O, which reflected the chondroprotective effect of the treatment.

### 4.8. Cell Culture Model

The human chondrosarcoma cell line (SW1353; ATCC, HTB-94, passage 6–10) was used to investigate the effects of KP extract on gene expression levels. The cells were authenticated by DiagCor Bioscience Incorporation Limited (Hong Kong, China). They were grown in DMEM with 10% FBS in a humidified incubator with 5% CO_2_ at 37 °C. Cells at 80% confluence were seeded into 6-well plates and starved for 24 h prior to pretreat with a combination of IL-1β 2 ng/mL and IL-17A 4 ng/mL for the inflammatory stimulation. The KP extract (1, 3, and 10 μg/mL), standard active compounds (D = 3.3 μg/mL, T = 2.6 μg/mL, P = 2.2 μg/mL, and D + T + P = 3.3 + 2.6 + 2.2 μg/mL), and diacerein (50 μM) were co-treated with the combined cytokines for 24 h. In addition, pretreatment with TNF-α 10 ng/mL was performed to trigger cell death-associated genes followed by co-treatment with the KP extract until 24 h for gene analysis and 48 h for flow cytometric analysis. LDH levels in culture media were measured using LDH assay, as previously described [45] (Figure S4). Additionally, the IC_50_ of KP extract in SW1353 cells, human articular chondrocyte, and porcine articular chondrocyte by MTT assay were 70.4, 43.8, and 162.3 μg/mL (Figure S5).

### 4.9. Real-Time Reverse Transcription Polymerase Chain Reaction (RT-PCR)

The cells were lysed, and total RNA was extracted by using an RNAspin mini kit (GE Healthcare). ReverTra Ace qPCR RT master mix (TOYOBO, Osaka, Japan) was used to synthesize cDNA from 1 μg of RNA. Real-time PCR was performed on a 7500 Fast Real-time PCR system (Applied Biosystems, Darmstadt, Germany), and SensiFast SYBR Lo-ROX reagent (Bioline, London, UK) was used. The specific primer sequences were designed by using the NCBI Primer-BLAST tool (Table S1). Data were calculated by using the 2^−ΔΔ*C*t^ method and normalized to the *GAPDH* reference gene.

### 4.10. Western Blot Analysis

SW1353 was seeded into a 25 cm^2^ culture flask at 80% confluence and starved for 24 h. Pretreatments with KP extract (10 μg/mL), standard active compounds (D = 3.3 μg/mL, T = 2.6 μg/mL, P = 2.2 μg/mL, and D + T + P = 3.3 + 2.6 + 2.2 μg/mL), and signaling inhibitors, including BAY11-7082 (NF-κB inhibitor; 20 µM), SB203580 (p38 inhibitor; 1 µM), SP600125 (JNK inhibitor; 10 µM), and U0126 (ERK inhibitor; 5 µM), were performed for 2 h. The cells were then co-treated at final concentrations of IL-1β 2 ng/mL and IL-17A 4 ng/mL for 25–30 min and immediately collected in radioimmunoprecipitation assay lysis buffer. The proteins in the cell lysates were separated by using sodium dodecyl sulfate polyacrylamide gel electrophoresis and electro-transferred to nitrocellulose membranes. Signaling molecules were targeted by specific antibodies (Table S2) and developed by using SuperSignal West Femto Maximum Sensitivity Substrate (Thermo Fisher Scientific, Waltham, MA, USA). Quantity One (Bio-Rad, Hercules, CA, USA) was used to capture the images without adjustments. The phosphorylated and total forms of each molecule were quantified on the same membrane, which was deprobed by using a stripping buffer (Thermo Fisher Scientific, Waltham, MA, USA). TotalLab TL120 v2009 software (TotalLab Ltd., Newcastle-Upon-Tyne, UK) was used to measure densities of the target bands.

### 4.11. Flow Cytometric Analysis

SW1353 cells with 80% confluence in 24-well plates were pretreated with TNF-α 50 ng/mL for 2 h and then co-treated with KP extract until 48 h. Cells were trypsinized and stained by using an Annexin-V-FLUOS staining kit (Roche Applied Science, Indianapolis, IN, USA) according to the manufacturer’s instructions. Apoptotic cells were counted by using a BD FACSCanto II Flow Cytometer (BD Biosciences Systems, San Jose, CA, USA). A cell death positive control was prepared in 2.5% DMSO.

### 4.12. Statistical Analysis

The data were expressed as the mean ± SEM or SD of two or three independent experiments that were conducted in duplicate or triplicate. The statistical analyses were determined by using one-way analysis of variance followed by the post hoc Tukey multiple comparison test or Fisher’s test. Values of *p* ≤ 0.05 were taken to be indicative of statistical significance. SPSS 22.0 version was used for statistical analysis.

## Figures and Tables

**Table 1:** Histological grading of synovial inflammation in the hind paw joint of the experimental rats using Gynther’s system [14].

Group	Synovial Lining Layers (Point)	Vascularity (Point)	Inflammatory Cells (Point)
Normal	0.0 ± 0.0	0.3 ± 0.3	0.7 ± 0.7
Arthritis	1.5 ± 0.3 *	1.8 ± 0.5 *	6.8 ± 2.0 *
Indo	1.3 ± 0.3 *	0.6 ± 0.2 #	2.6 ± 0.7 #
KP150	0.4 ± 0.2	0.6 ± 0.2 #	1.6 ± 0.7 #
KP300	0.4 ± 0.2	0.8 ± 0.2	1.6 ± 0.4 #

The induced rats after feeding with 3 mg/kg/day of indomethacin (Indo), 150 mg/kg/day of KP extract (KP150) or 300 mg/kg/day of KP extract (KP300) for 28 days were observed on histopathological assessments of synovitis in the blinded samples. Results represent mean ± SEM of 3 rats (normal group) or 6 rats (treated groups). One-way ANOVA followed by the post hoc Tukey multiple comparison test was used to analyze the statistical difference (* *p* < 0.05 compared to the normal group and # *p* < 0.05 compared to the arthritis group).

**Table 2:** The biological parameters of the experimental rats.

	Normal	Arthritis	Indo	KP150	KP300
**Organ Weights**					
Liver (g)	5.20 ± 0.19	4.94 ± 0.41	5.18 ± 0.28	4.38 ± 0.10	5.11 ± 0.17
Spleen (g)	0.42 ± 0.01	0.41 ± 0.02	0.60 ± 0.07 *	0.37 ± 0.01	0.42 ± 0.01
Kidney (g)	1.37 ± 0.00	1.33 ± 0.04	1.24 ± 0.03	1.17 ± 0.02 *	1.35 ± 0.06
Thymus (g)	0.22 ± 0.03	0.19 ± 0.02	0.18 ± 0.02	0.18 ± 0.02	0.23 ± 0.02
**Hematological Parameters**					
White blood cell (10^3^/μL)	2.49 ± 0.42	2.46 ± 0.30	4.09 ± 0.49	2.55 ± 0.42	3.00 ± 0.39
Red blood cell (10^6^/μL)	7.03 ± 0.12	7.09 ± 0.14	5.29 ± 0.34 *	7.40 ± 0.14	7.32 ± 0.12
Hemoglobin (g/dL)	13.2 ± 0.2	13.5 ± 0.2	10.3 ± 0.6 *	13.9 ± 0.2	13.7 ± 0.2
Hematocrit (%)	40.5 ± 0.7	40.9 ± 0.6	33.1 ± 1.7 *	41.8 ± 0.5	41.6 ± 0.7
Platelet (10^3^/μL)	679 ± 61	695 ± 18	1044 ± 147	677 ± 20	571 ± 38
**Blood Chemistry Tests**					
Glucose (mg/dL)	130 ± 8	110 ± 9	84 ± 11	116 ± 7	122 ± 12
Aspartate aminotransferase (U/L)	110 ± 2	122 ± 15	122 ± 12	120 ± 11	106 ± 22
Alanine aminotransferase (U/L)	44 ± 5	48 ± 2	33 ± 3	37 ± 1	46 ± 3
Alkaline phosphatase (U/L)	106 ± 5	113 ± 5	139 ± 34	114 ± 5	131 ± 10
Blood urea nitrogen (mg/dL)	16.1 ± 1.2	19.6 ± 2.0	18.9 ± 1.0	16.3 ± 0.8	17.8 ± 1.0
Creatinine (mg/dL)	0.56 ± 0.03	0.58 ± 0.02	0.54 ± 0.01	0.54 ± 0.01	0.54 ± 0.01

The induced rats after feeding with 3 mg/kg/day of indomethacin (Indo), 150 mg/kg/day of KP extract (KP150) or 300 mg/kg/day of KP extract (KP300) for 28 days were measured the organ weights, hematological parameters, and blood chemistry parameters immediately after euthanasia. Results represent mean ± SEM of 3 rats (normal group) or 6 rats (treated groups). One-way ANOVA followed by the post hoc Tukey multiple comparison test was used to analyze the statistical difference (* *p* < 0.05 compared to normal group).

## Data Availability

The data presented in this study are available on request from the corresponding author.

## Supplementary Materials

The following are available online, Table S1: The human primer sequences used for real-time RT-PCR, Table S2: The commercial antibodies used for western blot analysis, Figure S1: HPLC chromatograms of KP extract, Figure S2: Effects of KP extract on changes of rat body weight, Figure S3: Effects of KP extract on cell cycle in TNF-α-induced SW1353 cells for 48 h, Figure S4: Effects of KP extract on LDH release in IL-1β and IL-17A-induced SW1353 cells for 24 h, Figure S5: Effects of KP extract on cell viability of SW1353 cell, Figure S6: Full-length blot images of IKK, IκB, p65, and β-actin, Figure S7; Full-length blot images of JNK, ERK, p38, and β-actin, Figure S8; The chemical structure of standard compounds.

## Sample Availability

Samples of the compounds are not available from the authors.
