# Supplementary material for: Kaempferia parviflora Extract Alleviated Rat Arthritis, Exerted Chondroprotective Properties In Vitro, and Reduced Expression of Genes Associated with Inflammatory Arthritis
Source: Molecules. 2021 Mar 11;26(6):1527. doi: 10.3390/molecules26061527 (PMC8000004; doi:10.3390/molecules26061527)
Supplement: Supplementary file 1 [file molecules-26-01527-s001.pdf]

SUPPLEMENTARY FIGURES AND TABLES

Table S1. The human primer sequences used for real-time RT-PCR.

Genes	Accession number	Primer sequences
GAPDH	NM_002046.5	F: 5′-GAAGGTGAAGGTCGGAGTC-3′<br>R: 5′-GAAGATGGTGATGGGATTTC-3′
IL1B	NM_000576.2	F: 5′-CCTGTCCTGCGTGTTGAAAGA-3′<br>R: 5′-GGGAACTGGGCAGACTCAAA-3′
IL6	NM_000600.4	F: 5′-GGTACATCCTCGACGGCATCT-3′<br>R: 5′-GTGCCTCTTTGCTGCTTTCAC-3′
TNF	NM_000594.3	F: 5′-CCCCAGGGACCTCTCTCTAATC-3′<br>R: 5′-GGTTTGCTACAACATGGGCTACA-3′
MMP13	NM_002427.3	F: 5′-AAACGCCAGACAAATGTGACC-3′<br>R: 5′-AGCATCAATACGGTTGGGAAG-3′
ZIP8	NM_022154.5	F: 5′-TCCTGCACCTTGTCTCTCCT-3′<br>R: 5′-TTGTCGAGTGCTCATCCCTG-3′
BCL2	NM_000633.2	F: 5′-TGTGTGTGGAGAGCGTCAA-3′<br>R: 5′-GACAGCCAGGAGAAATCAAACAG-3′
BAX	NM_001291428.1	F: 5′-AGCAGATCATGAAGACAGGGG-3′<br>R: 5′-ACACTCGCTCAGCTTCTTGG-3′
RIPK1	NM_003804.5	F: 5′-CGTAAACTGGGCTTCACACAG-3′<br>R: 5′-CCTTCCCTCATCACCCACTTT-3′
RIPK3	NM_006871.3	F: 5′-CAGTGTGCAACAGGCAGAAC-3′<br>R: 5′-TCAGTCCTTCTAAGCCGGGA-3′

Table S2. The commercial antibodies used for western blot analysis.

**Primary antibodies**			
**Species**	**Targets**	**Dilution**	**Sources (catalogue number)**
Rabbit	Phospho-IKKα/β	1:1000	Cell Signaling Technology (2697)
Mouse	IKKα	1:1000	Cell Signaling Technology (11930)
Rabbit	IKKβ	1:1000	Cell Signaling Technology (8943)
Rabbit	Phospho-IκBα	1:1000	Cell Signaling Technology (2859)
Mouse	IκBα	1:1000	Cell Signaling Technology (4814)
Rabbit	Phospho-NF-κB p65	1:1000	Cell Signaling Technology (3033)
Rabbit	NF-κB p65	1:1000	Cell Signaling Technology (8242)
Rabbit	Phospho-SAPK/JNK	1:1000	Cell Signaling Technology (4668)
Rabbit	SAPK/JNK	1:1000	Cell Signaling Technology (9252)
Rabbit	Phospho-p44/42 MAPK (Erk1/2)	1:1000	Cell Signaling Technology (4370)
Rabbit	p44/42 MAPK (Erk1/2)	1:1000	Cell Signaling Technology (4695)
Rabbit	Phospho-p38 MAPK	1:1000	Cell Signaling Technology (4511)
Rabbit	p38 MAPK	1:1000	Cell Signaling Technology (8690)
Mouse	β-actin	1:500	BioLegend (BIOL-643802)
**Secondary antibodies**			
Goat	Rabbit IgG	1:2000	Cell Signaling Technology (7074)
Horse	Mouse IgG	1:2000	Cell Signaling Technology (7076)

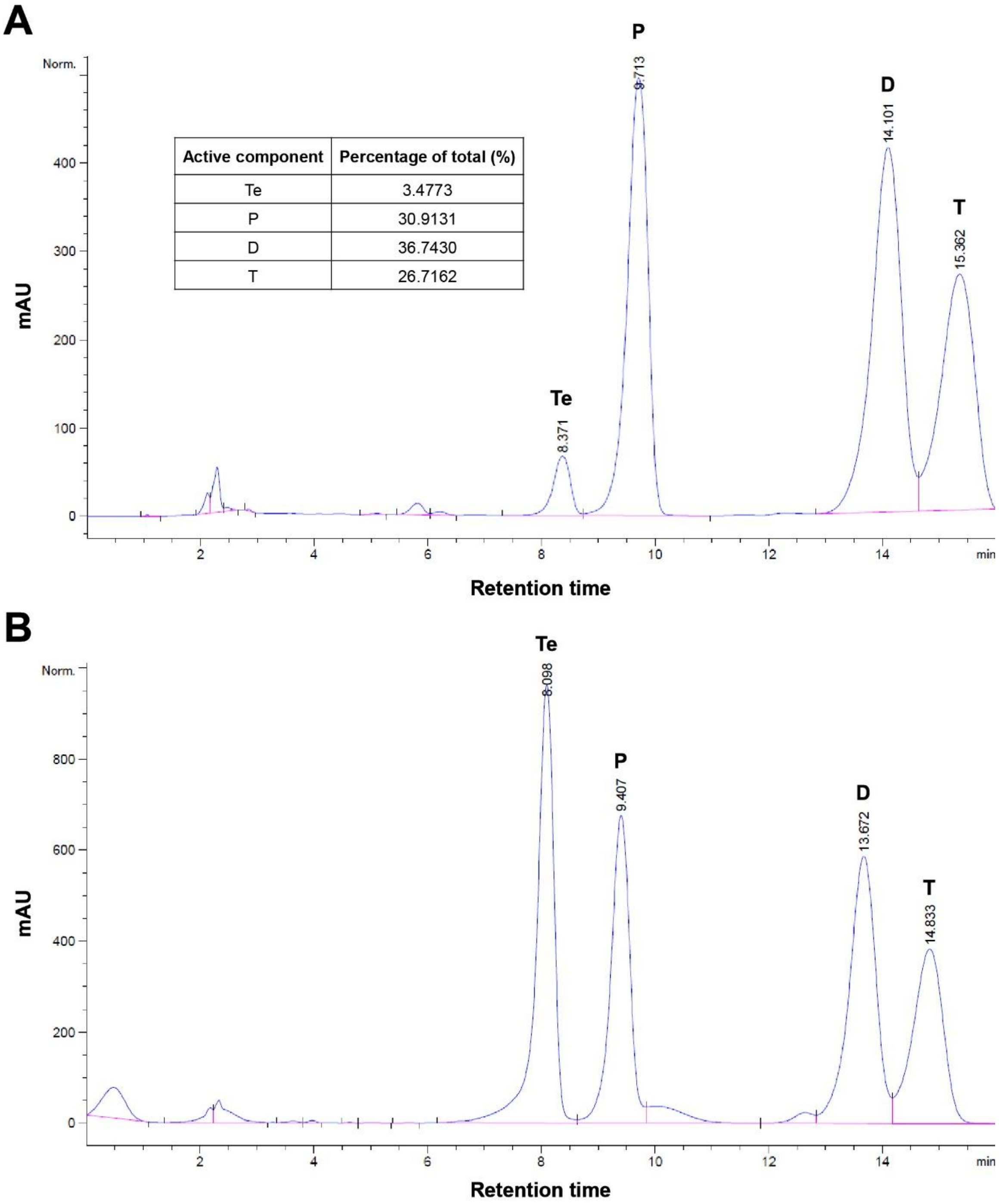

Active component	Percentage of total (%)
Te	3.4773
P	30.9131
D	36.7430
T	26.7162

Figure S1. HPLC chromatograms of KP extract (**A**) and standards of methoxyflavones (**B**). “Te” = 5,7,3′,4′-tetramethoxyflavone, “P” = 3,5,7,3′,4′-pentamethoxyflavone, “D” = 5,7-dimethoxyflavone, and “T” = 5,7,4′-trimethoxyflavone.

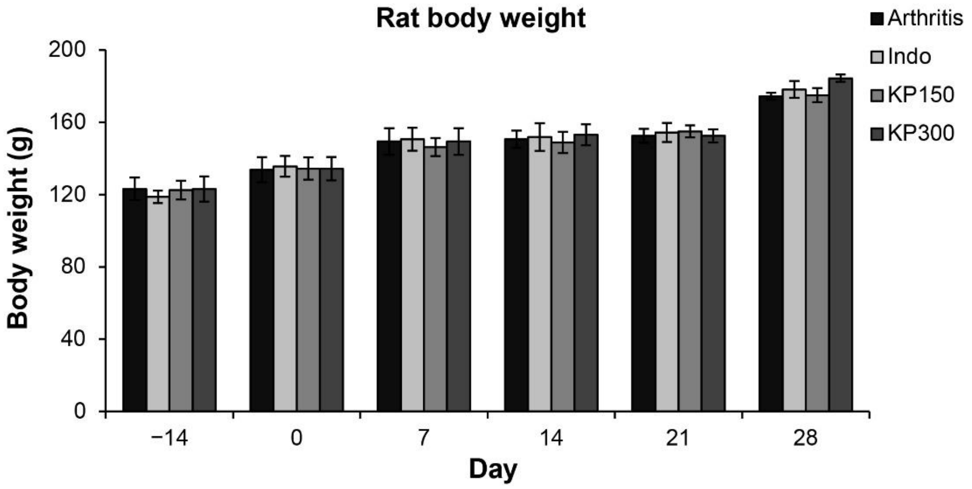

Figure S2. Effects of KP extract on changes of rat body weight. “Arthritis” = arthritis rats control, “Indo” = arthritis rats treated with indomethacin at 3 mg/kg/day, “KP 150” and “KP 300” = arthritis rats treated with *Kaempferia parviflora* extract at 150 and 300 mg/kg/day. Results represent mean ± SEM of 6 rats per group.

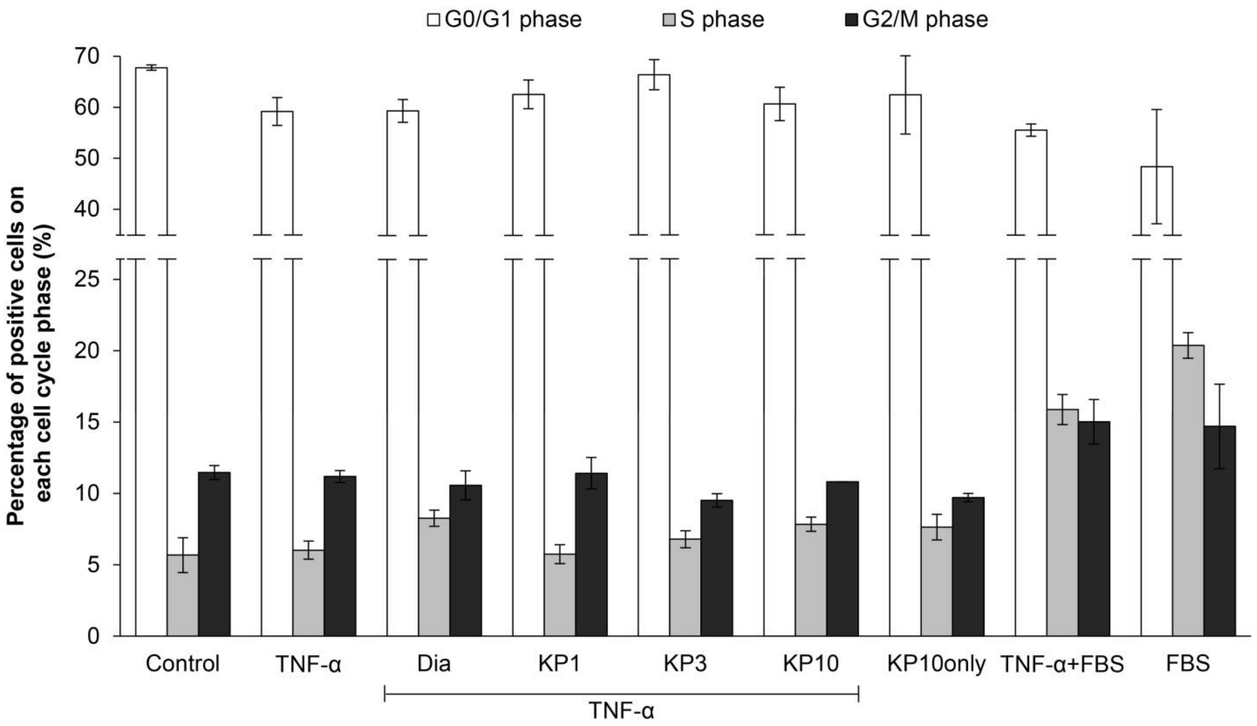

Figure S3. Effects of KP extract on cell cycle in TNF-α-induced SW1353 cells for 48 h. 10% fetal bovine serum (FBS) was used as a positive control of cell cycle induction. Cells were stained with PI and evaluated by flow cytometer. Data are expressed as mean ± SD of duplicate independent experiments. “Dia” = diacerein (50 μM), and “KP” = *Kaempferia parviflora* extract at indicated concentration (1, 3, and 10 μg/mL).

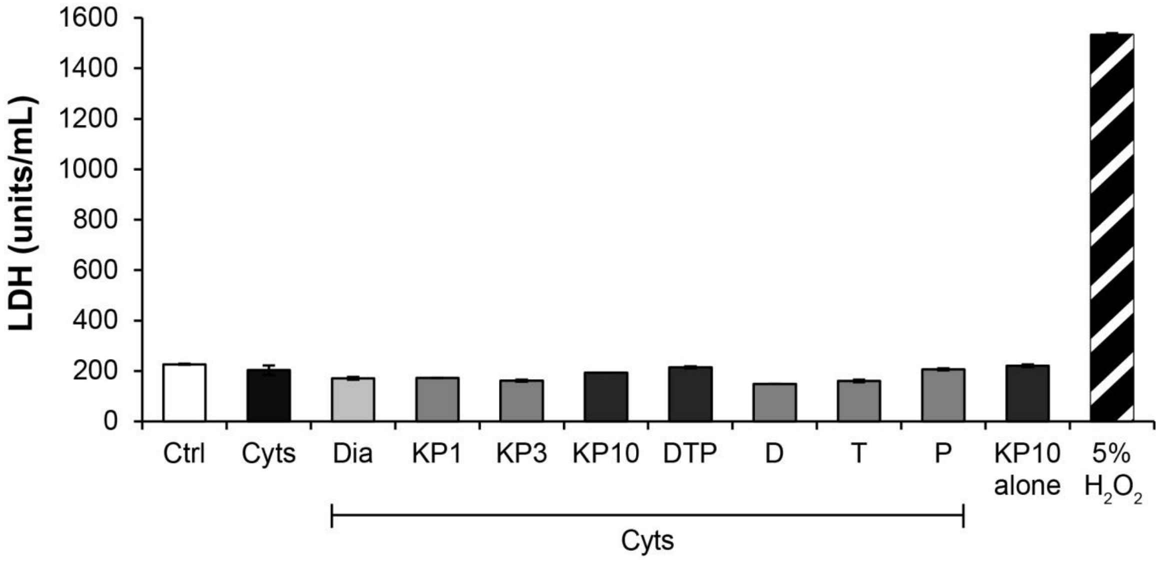

Figure S4. Effects of KP extract on LDH release in IL-1β and IL-17A-induced SW1353 cells for 24 h. 5% H_2O_2 was used as a positive control of LDH induction. Data are expressed as mean ± SD of triplicate independent experiments. "Ctrl" = control, "Cyts" = cytokines (a combination of 2 ng/mL IL-1β and 4 ng/mL IL-17A), "Dia" = diacerein (50 μM), "KP" = *Kaempferia parviflora* extract at indicated concentration (1, 3, and 10 μg/mL), "D" = 5,7-dimethoxyflavone (DMF; 3.3 μg/mL), "T" = 5,7,4′-trimethoxyflavone (TMF; 2.6 μg/mL), "P" = 3,5,7,3′,4′-pentamethoxyflavone (PMF; 2.2 μg/mL), and "DTP" = a mixture of the three major compounds (DMF: TMF: PMF = 3.3: 2.6: 2.2 μg/mL).

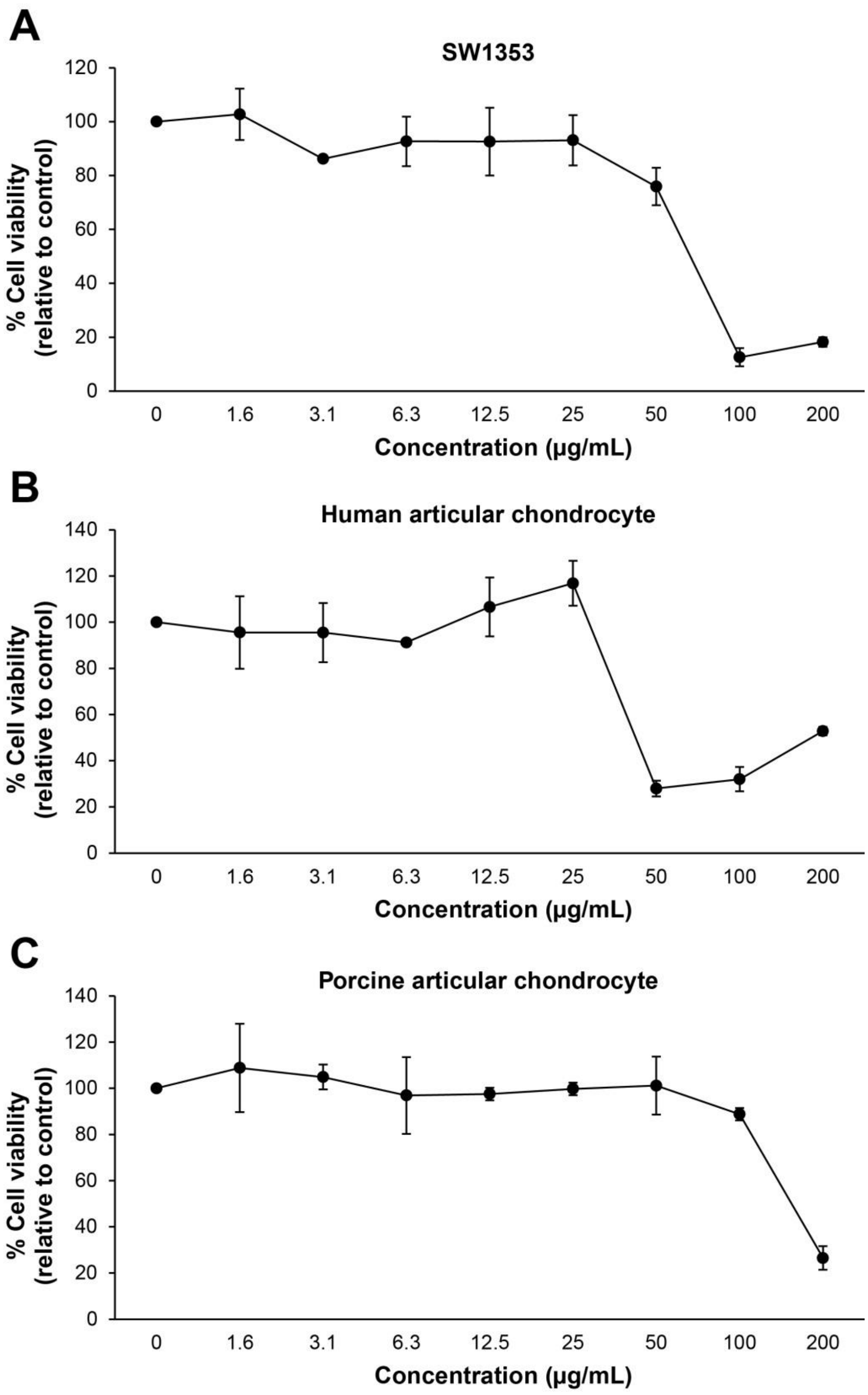

Figure S5. Effects of KP extract on cell viability of SW1353 cell (**A**), human articular chondrocyte (**B**), and porcine articular chondrocyte (**C**) by MTT assay at concentrations ranging from 0 to 200 μg/mL for 24 h. Data are expressed as mean ± SD of duplicate independent experiments.

A

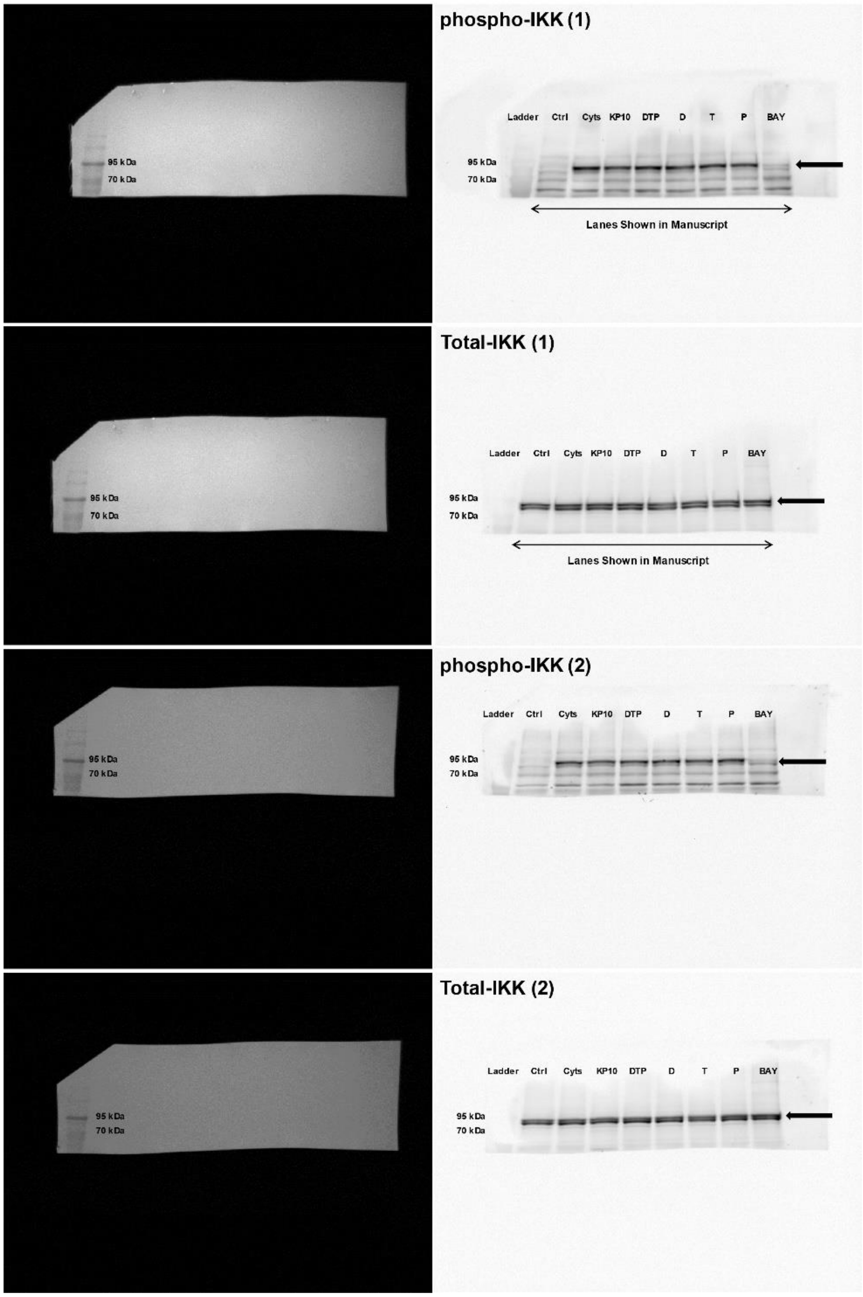
phospho-IKK (1)
Ladder Ctrl Cyts KP10 DTP D T P BAY
95 kDa
70 kDa
Lanes Shown in Manuscript
Total-IKK (1)
Ladder Ctrl Cyts KP10 DTP D T P BAY
95 kDa
70 kDa
Lanes Shown in Manuscript
phospho-IKK (2)
Ladder Ctrl Cyts KP10 DTP D T P BAY
95 kDa
70 kDa
Total-IKK (2)
Ladder Ctrl Cyts KP10 DTP D T P BAY
95 kDa
70 kDa

B

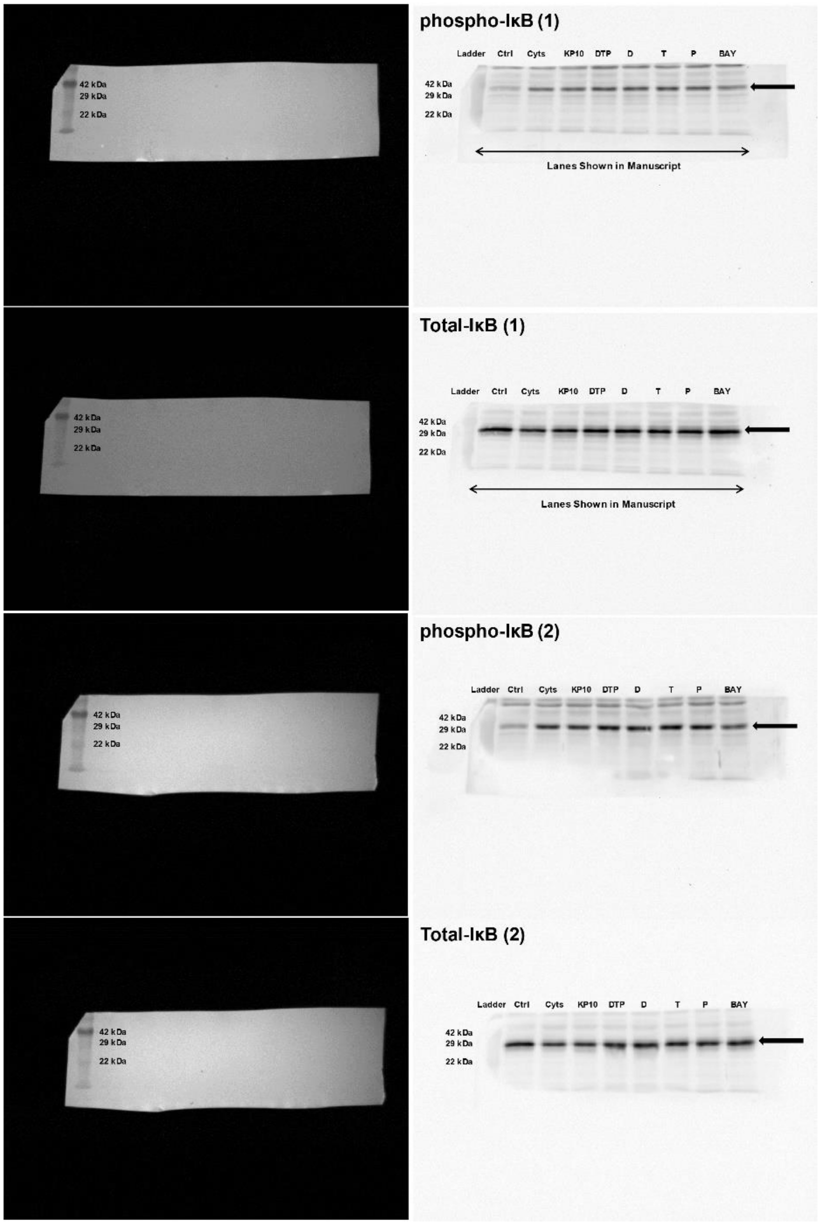
42 kDa
29 kDa
22 kDa
phospho-IκB (1)
Ladder Ctrl Cyts KP10 DTP D T P BAY
42 kDa
29 kDa
22 kDa
Lanes Shown in Manuscript
42 kDa
29 kDa
22 kDa
Total-IκB (1)
Ladder Ctrl Cyts KP10 DTP D T P BAY
42 kDa
29 kDa
22 kDa
Lanes Shown in Manuscript
42 kDa
29 kDa
22 kDa
phospho-IκB (2)
Ladder Ctrl Cyts KP10 DTP D T P BAY
42 kDa
29 kDa
22 kDa
42 kDa
29 kDa
22 kDa
Total-IκB (2)
Ladder Ctrl Cyts KP10 DTP D T P BAY
42 kDa
29 kDa
22 kDa

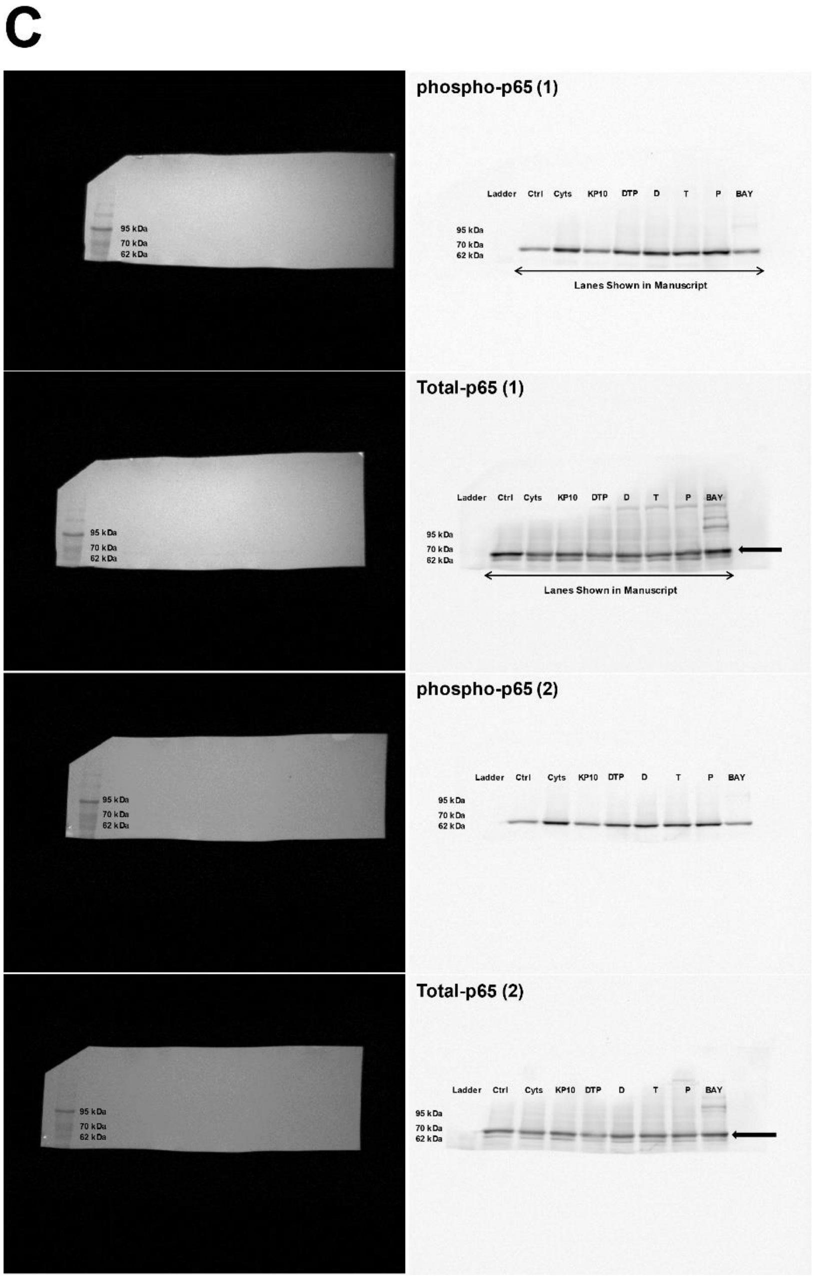
C
phospho-p65 (1)
Ladder Ctrl Cyts KP10 DTP D T P BAY
95 kDa
70 kDa
62 kDa
Lanes Shown in Manuscript
Total-p65 (1)
Ladder Ctrl Cyts KP10 DTP D T P BAY
95 kDa
70 kDa
62 kDa
Lanes Shown in Manuscript
phospho-p65 (2)
Ladder Ctrl Cyts KP10 DTP D T P BAY
95 kDa
70 kDa
62 kDa
Total-p65 (2)
Ladder Ctrl Cyts KP10 DTP D T P BAY
95 kDa
70 kDa
62 kDa

D

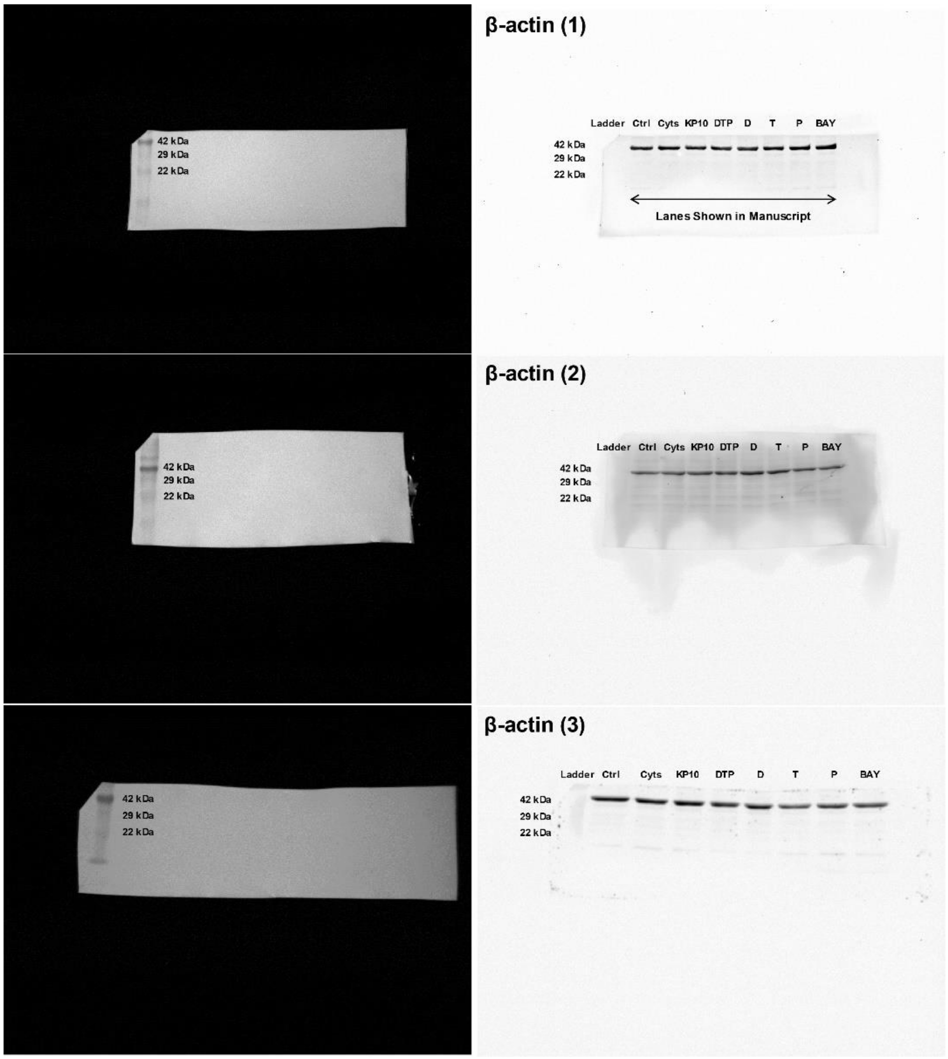

Figure S6. Full-length blot images of IKK (**A**), IκB (**B**), p65 (**C**), and β-actin (**D**). SW1353 cells were pretreated for 2 h with KP extract (10 μg/mL), 5,7-dimethoxyflavone (D; 3.3 μg/mL), 5,7,4′-trimethoxyflavone (T; 2.6 μg/mL), 3,5,7,3′,4′-pentamethoxyflavone (P; 2.2 μg/mL) and a mixture of the three major compounds (DTP), which equal to the estimated proportions in the KP extract at 10 μg/mL (D: T: P = 3.3: 2.6: 2.2 μg/mL), and signaling inhibitor, BAY11-7082 (BAY). The cells were then co-treated with IL-1β 2 ng/mL and IL-17A 4 ng/mL (Cyts) for 25–30 min. The untreated group was left as a control (Ctrl).

A

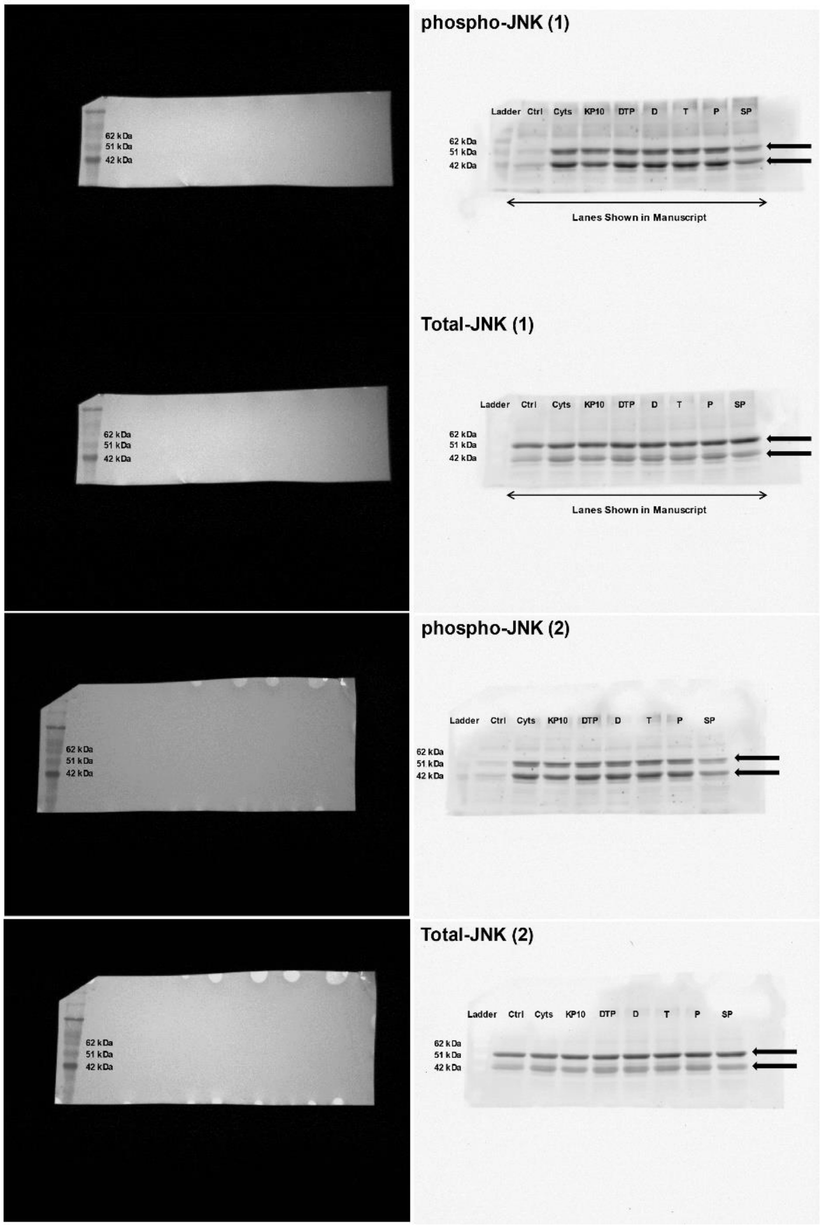
phospho-JNK (1)
Ladder Ctrl Cyts KP10 DTP D T P SP
62 kDa
51 kDa
42 kDa
Lanes Shown in Manuscript
Total-JNK (1)
Ladder Ctrl Cyts KP10 DTP D T P SP
62 kDa
51 kDa
42 kDa
Lanes Shown in Manuscript
phospho-JNK (2)
Ladder Ctrl Cyts KP10 DTP D T P SP
62 kDa
51 kDa
42 kDa
Total-JNK (2)
Ladder Ctrl Cyts KP10 DTP D T P SP
62 kDa
51 kDa
42 kDa

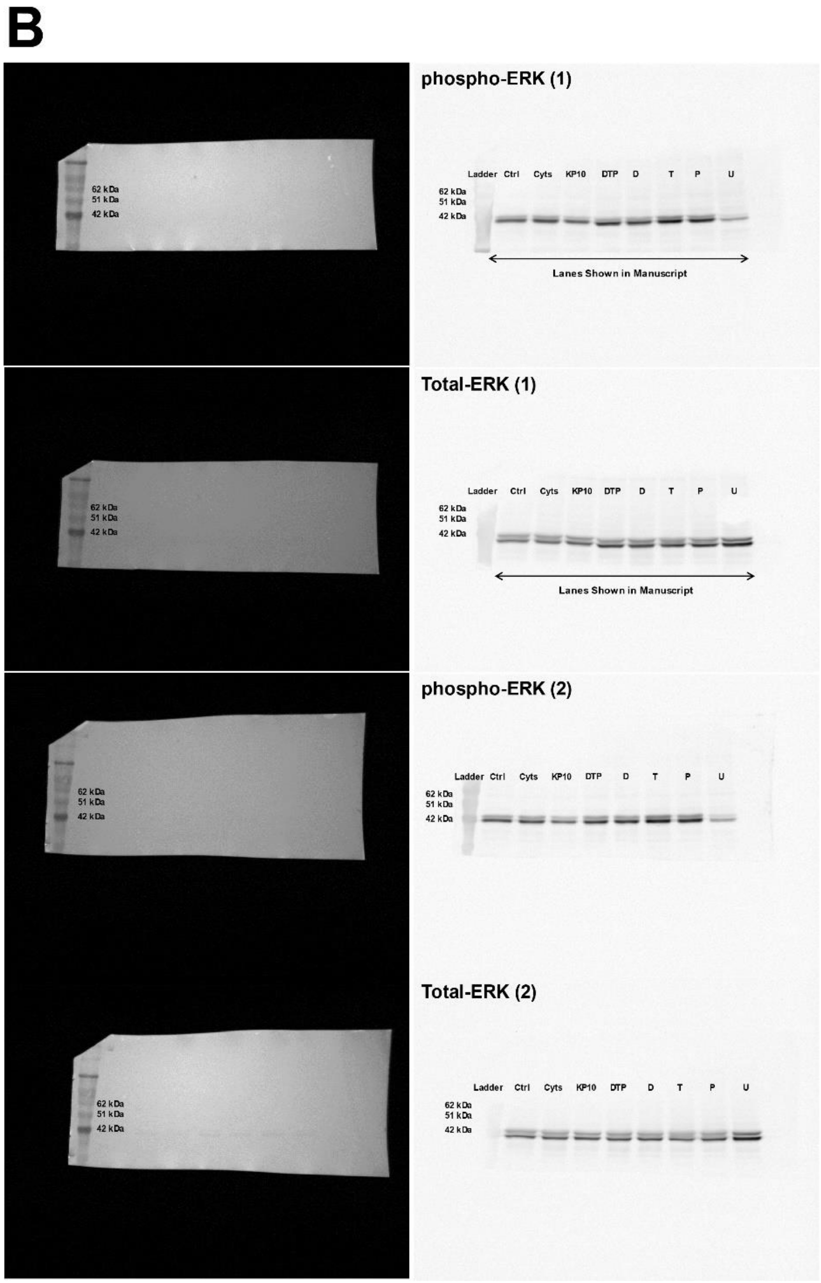
B
phospho-ERK (1)
Ladder Ctrl Cyts KP10 DTP D T P U
62 kDa
51 kDa
42 kDa
Lanes Shown in Manuscript
Total-ERK (1)
Ladder Ctrl Cyts KP10 DTP D T P U
62 kDa
51 kDa
42 kDa
Lanes Shown in Manuscript
phospho-ERK (2)
Ladder Ctrl Cyts KP10 DTP D T P U
62 kDa
51 kDa
42 kDa
Total-ERK (2)
Ladder Ctrl Cyts KP10 DTP D T P U
62 kDa
51 kDa
42 kDa

C

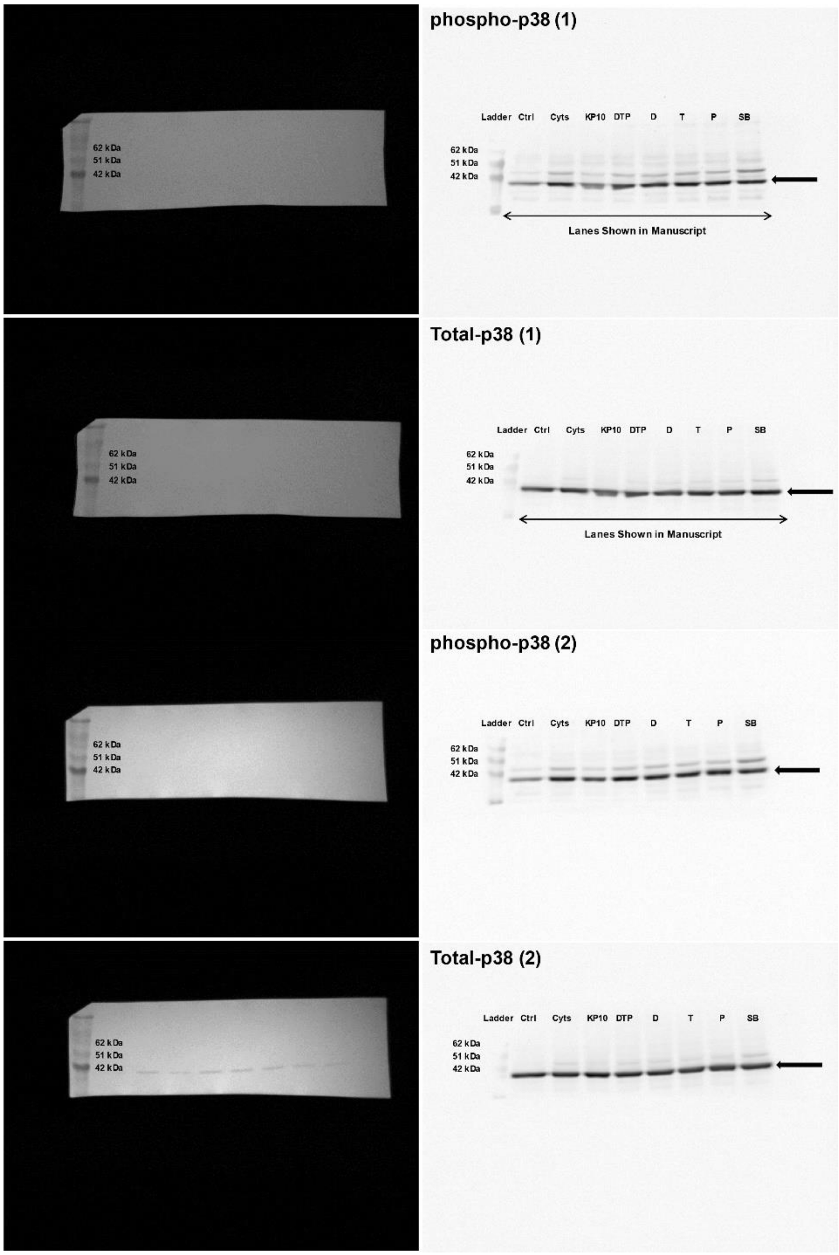
phospho-p38 (1)
Ladder Ctrl Cyts KP10 DTP D T P SB
62 kDa
51 kDa
42 kDa
Lanes Shown in Manuscript
Total-p38 (1)
Ladder Ctrl Cyts KP10 DTP D T P SB
62 kDa
51 kDa
42 kDa
Lanes Shown in Manuscript
phospho-p38 (2)
Ladder Ctrl Cyts KP10 DTP D T P SB
62 kDa
51 kDa
42 kDa
Total-p38 (2)
Ladder Ctrl Cyts KP10 DTP D T P SB
62 kDa
51 kDa
42 kDa

D

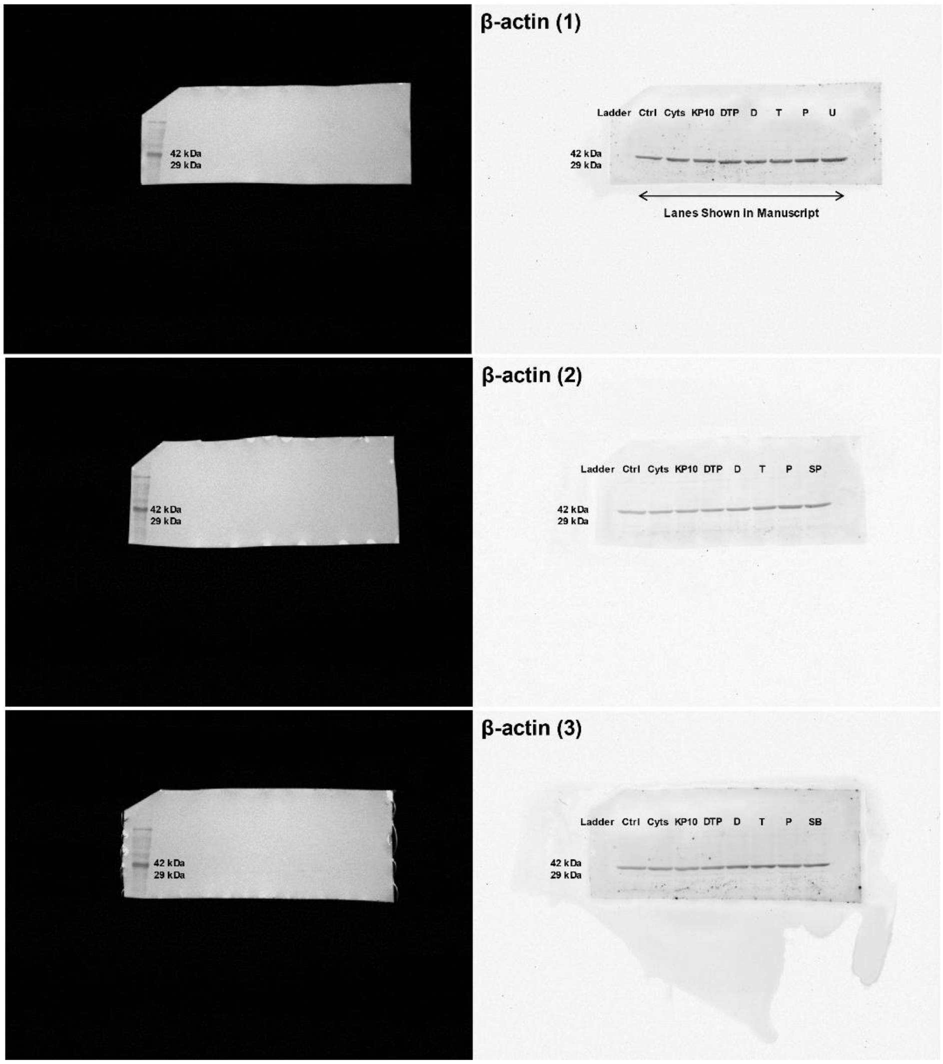

Figure S7. Full-length blot images of JNK (**A**), ERK (**B**), p38 (**C**), and β-actin (**D**). SW1353 cells were pretreated for 2 h with KP extract (10 µg/mL), 5,7-dimethoxyflavone (D; 3.3 µg/mL), 5,7,4′-trimethoxyflavone (T; 2.6 µg/mL), 3,5,7,3′,4′-pentamethoxyflavone (P; 2.2 µg/mL) and a mixture of the three major compounds (DTP), which equal to the estimated proportions in the KP extract at 10 µg/mL (D: T: P = 3.3: 2.6: 2.2 µg/mL), and signaling inhibitors, including SB203580 (SB), SP600125 (SP), and U0126 (U). The cells were then co-treated with IL-1β 2 ng/mL and IL-17A 4 ng/mL (Cyts) for 25–30 min. The untreated group was left as a control (Ctrl).

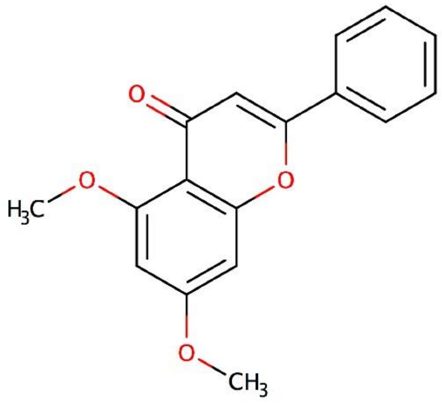

5,7-dimethoxyflavone (D)

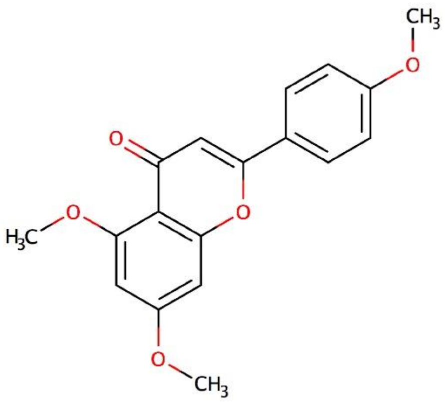

5,7,4'-trimethoxyflavone (T)

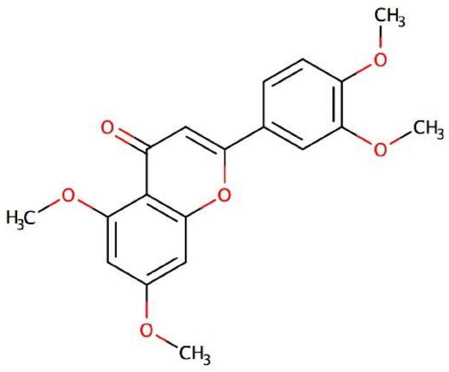

5,7,3',4'-tetramethoxyflavone (Te)

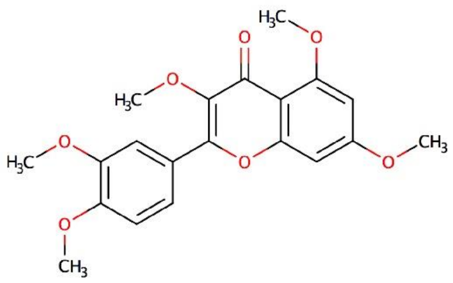

3,5,7,3',4'-pentamethoxyflavone (P)

Figure S8. The chemical structures of 5,7-dimethoxyflavone (D), 5,7,4'-trimethoxyflavone (T), 5,7,3',4'-tetramethoxyflavone (Te), and 3,5,7,3',4'-pentamethoxyflavone (P). The structures are available from the distributor website: https://indofinechemical.com/.
